# CDCA7-associated global aberrant DNA hypomethylation translates to localized, tissue-specific transcriptional responses

**DOI:** 10.1126/sciadv.adk3384

**Published:** 2024-02-09

**Authors:** Maja Vukic, Jihed Chouaref, Veronica Della Chiara, Serkan Dogan, Fallon Ratner, Jenna Z. M. Hogenboom, Trevor A. Epp, Kallayanee Chawengsaksophak, Kelly K. D. Vonk, Cor Breukel, Yavuz Ariyurek, David San Leon Granado, Susan L. Kloet, Lucia Daxinger

**Affiliations:** ^1^ Department of Human Genetics, Leiden University Medical Center, Leiden, Netherlands.; ^2^ Laboratory of Cell Differentiation, Institute of Molecular Genetics of the Czech Academy of Sciences, Prague, Czech Republic.; ^3^ CZ-OPENSCREEN, Institute of Molecular Genetics of the Czech Academy of Sciences, Prague, Czech Republic.; ^4^ Leiden Genome Technology Center, Department of Human Genetics, Leiden University Medical Center, Leiden, Netherlands.

## Abstract

Disruption of cell division cycle associated 7 (CDCA7) has been linked to aberrant DNA hypomethylation, but the impact of DNA methylation loss on transcription has not been investigated. Here, we show that CDCA7 is critical for maintaining global DNA methylation levels across multiple tissues in vivo. A pathogenic *Cdca7* missense variant leads to the formation of large, aberrantly hypomethylated domains overlapping with the B genomic compartment but without affecting the deposition of H3K9 trimethylation (H3K9me3). CDCA7-associated aberrant DNA hypomethylation translated to localized, tissue-specific transcriptional dysregulation that affected large gene clusters. In the brain, we identify CDCA7 as a transcriptional repressor and epigenetic regulator of clustered *protocadherin* isoform choice. Increased *protocadherin* isoform expression frequency is accompanied by DNA methylation loss, gain of H3K4 trimethylation (H3K4me3), and increased binding of the transcriptional regulator CCCTC-binding factor (CTCF). Overall, our in vivo work identifies a key role for CDCA7 in safeguarding tissue-specific expression of gene clusters via the DNA methylation pathway.

## Teaser

CDCA7-mediated DNA methylation facilitates tissue-specific expression of gene clusters.

## INTRODUCTION

DNA methylation is a well-known epigenetic modification associated with transcriptional repression and is pivotal for normal development (*1*). Genome-wide aberrant methylation is increasingly being reported in developmental disorders (*2*). Links between DNA methylation alterations and gene expression changes have been widely explored; however, the relation, significance, and functional relevance of these alterations remain poorly understood.

Recessive missense mutations in *CDCA7* (*cell division cycle associated 7*) can cause immunodeficiency, centromeric instability, facial anomalies (ICF) syndrome (*3*), a genetically heterogeneous disorder characterized by reduced levels or absence of antibodies, facial dysmorphisms, and neurodevelopmental delay (*4*). In the blood of patients with ICF carrying *CDCA7* missense mutations, aberrant DNA hypomethylation of CpG-poor genomic regions and (peri)centromeric satellite repeat sequences has been observed (*3*, *5*). CDCA7 knockdown and knockout studies showed that CDCA7 disruption reduces DNA methylation levels at (peri)centromeric satellite repeats using mouse (*3*, *6*) and human cell lines (*7*). We recently reported an association between *CDCA7* expression levels and trans methylation changes at transcriptionally repressed sites in the blood of healthy individuals (*8*). In vitro studies identified an interaction between CDCA7 and the chromatin remodeler helicase lymphoid-specific (HELLS/Lsh) in HEK293 cells (*7*) and *Xenopus* egg extract (*9*). When mutated in humans, HELLS also causes ICF syndrome (*3*), and patients with CDCA7 and HELLS ICF show overlapping DNA methylation defects in the blood (*5*). Thus, several lines of evidence indicate that CDCA7 is a previously unrecognized factor required for DNA methylation regulation. However, how ICF3 pathogenic CDCA7-associated methylation alterations translate to gene expression changes in vivo has not been investigated.

In this study, we generated mice carrying an ICF syndrome causing CDCA7 missense variant and used the *Cdca7^G305V^* mouse model as a tool to comprehensively investigate the epigenetic and transcriptional consequences of CDCA7 disruption in vivo. Overall, our data uncover that CDCA7 is a transcriptional repressor with a role in safeguarding tissue-specific expression of gene clusters via DNA methylation.

## RESULTS

### An ICF3 pathogenic *Cdca7* missense mutation disrupts CDCA7 association with chromatin in vivo

**Fig. 1.**
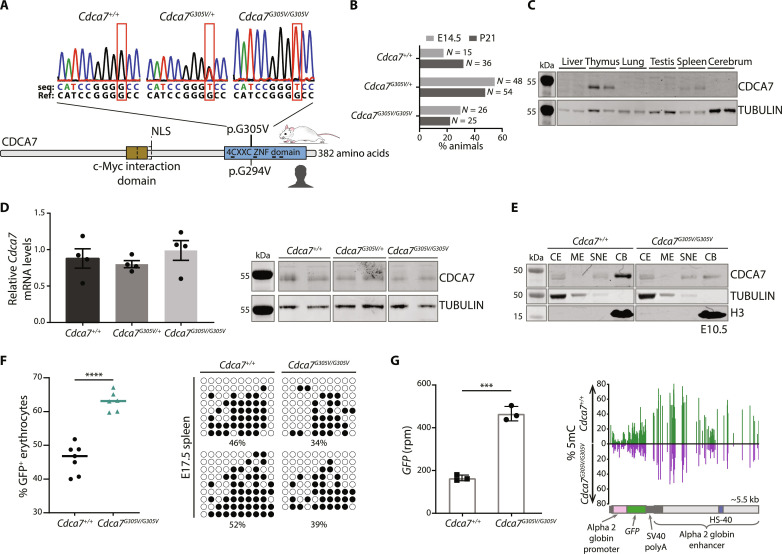
An ICF3-relevant *Cdca7* missense mutation disrupts CDCA7 association with chromatin in vivo. (**A**) Top: Representative Sanger sequencing traces from wild type (WT) (*Cdca7^+/+^*), heterozygotes (*Cdca7^G305V/+^*), and homozygotes (*Cdca7^G305V/G305V^*) depicting the c.914G > T mutation, and reference sequence for comparison. Bottom: Schematic of CDCA7 protein and the corresponding ICF3 patient mutation. (**B**) Bar chart showing the percentage of animals and their genotypes at mid-gestation (E14.5) and at weaning (P21). *N* = number of animals, collected from 10 (E14.5) and 14 litters (P21), respectively. (**C**) Western blot showing CDCA7 protein levels in different tissues at P21. TUBULIN was used as a loading control; two biological replicates per genotype. (**D**) Left: Quantitative reverse-transcriptase polymerase chain reaction (RT-qPCR) of relative *Cdca7* mRNA levels in P21 spleen (normalized to β-*actin*; *n* = 4 biological replicates; data are presented as mean with black dots as individual values; error bar = SEM). Right: Western blot showing CDCA7 protein in P21 spleen; two biological replicates per genotype; TUBULIN was used as a loading control. (**E**) Western blot showing CDCA7 protein levels in cytoplasmic extract (CE), membrane extract (ME), soluble nuclear extract (SNE), and chromatin-bound fraction (CB) isolated from E10.5 (4 pooled embryos per genotype). TUBULIN and H3 were used as loading controls. (**F**) Left: Percentage green fluorescent protein (GFP)–positive erythrocytes (TER-119–positive) in WT (*n* = 7) and *Cdca7^G305V^* homozygous (*n* = 6) embryonic spleens. ****P < 0.0001 (two-sided unpaired *t* test). Right: Transgene methylation levels in WT and *Cdca7^G305V^* homozygous embryonic spleen measured by bisulfite sequencing (filled circles – methylated cytosines; empty circle – unmethylated cytosines). (**G**) Left: *Gfp* mRNA levels determined by RNA sequencing (RNA-seq) and represented in rpm (reads per million) [data are presented as mean with black dots as individual values; error bars = SEM; ***P < 0.0001 (two-sided unpaired *t* test)]. Right: Schematic of the *GFP* transgene and methylation levels of WT and *Cdca7^G305V^* homozygous P21 spleen measured by whole-genome bisulfite sequencing (WGBS) (average of two biological replicates per genotype).

Using CRISPR-Cas9 genome editing, we introduced, in a *Line3/FVB* mouse line (*10*), the ICF3-causing *CDCA7* missense mutation c.914G > T; p.Gly294Val. This mutation changes glycine (G) 305 to valine (V) in the carboxyterminal 4-CXXC–type zinc finger (ZF) domain of mouse CDCA7. Successful editing was verified by Sanger sequencing, and no mutations were found in putative CRISPR off-target sites (Fig. 1A and fig. S1A). Therefore, we designated this mouse line as *Cdca7^G305V^*.

We first assessed the viability of *Cdca7^G305V^* mutants. We found that offspring of all genotypes from heterozygous intercrosses were present at normal Mendelian frequencies at mid-gestation [embryonic day 14.5 (E14.5)] and at postnatal day 21 (P21) (Fig. 1B and fig. S1B). While, at P21, *Cdca7^G305V^* homozygotes did not show overt phenotypes, we observed that homozygous females, on average, weighed less than wild type (WT) (*t* test, *P* < 0.0013) and heterozygotes (*t* test, *P* < 0.0005), while no differences in variance (*F* test, *P* < 0.39 and *P* < 0.9, respectively) were found (fig. S1C). However, in this study, we did not pursue any further in-depth phenotyping.

We then measured CDCA7 expression at P21 in tissues representing different germ layers by Western blot and quantitative reverse-transcriptase polymerase chain reaction (RT-qPCR) and found higher CDCA7 levels in proliferative tissues (spleen and thymus) of WT animals (Fig. 1C and fig. S1D). We detected no statistically significant differences in mRNA and no notable differences in protein levels in *Cdca7^G305V^* homozygotes when compared to WT and heterozygous littermates (Fig. 1D and fig. S1E), indicating that the G305V substitution does not influence CDCA7 levels in spleen and thymus. Next, we performed cellular fractionation assays on E10.5 embryo lysates from WT and *Cdca7^G305V^* homozygotes to determine the localization of CDCA7 in the cell. In WT, we detected CDCA7 protein in both the cytoplasm and the nucleus with the highest levels in the chromatin-bound fraction. In *Cdca7^G305V^* homozygotes, we observed lower amounts of CDCA7 protein in the chromatin-bound fraction (Fig. 1E), indicating that the ICF3 pathogenic G305V substitution in the CXXC-ZF domain interferes with CDCA7 chromatin association in vivo.

### CDCA7 influences the variegated expression of a transgene array in the spleen via DNA methylation

We previously used *Line3* in an in vivo chemical mutagenesis screen to identify modifiers of transgene array silencing where mutants are classified according to the *Drosophila* position affect variegation nomenclature, into *Suppressors* or *Enhancers of variegation* [*Su(var)* or *E(var)*] based on their ability to increase or decrease the percentage of green fluorescent protein (GFP)-expressing erythrocytes, respectively (*10*). From this screen, DNA methyltransferase 3 beta (DNMT3B), DNMT1, and ubiquitin-like with PHD and ring finger domains 1 (UHRF1) have been recovered (*11*–*13*), demonstrating its suitability to identify key DNA methylation factors. Mutant alleles are referred to as *Modifiers of murine metastable epialleles Dominant* (*MommeD*) and, in the WT *Line3*, the GFP transgene array is expressed in a variegated manner in ~50% of red blood cells, a result of stochastic epigenetic silencing that occurs in cells of the same type (*10*). Flow cytometry analyses on embryonic spleen revealed a significant increase in the percentage of erythrocytes expressing GFP in *Cdca7^G305V^* homozygotes when compared to WT (average GFP values for WT = 46% and homozygotes = 63%), consistent with a *Su(var)* phenotype (Fig. 1F and fig. S2). Sanger bisulfite sequencing showed that increased GFP expression was accompanied by lower transgene DNA methylation levels in *Cdca7^G305V^* homozygotes (average methylation levels for WT = 49% and homozygotes ~37%) (Fig. 1F). We further found that increased *Gfp* mRNA levels [RNA sequencing (RNA-seq)] correlated with transgene hypomethylation (whole-genome bisulfite sequencing, WGBS; 5-methylcytosine, 5mC) in P21 *Cdca7^G305V^* mutant spleen (Fig. 1G), consistent with our finding in embryos. These results show that the *Cdca7^G305V^* missense mutation increases the probability of a red blood cell expressing GFP by altering transgene DNA methylation levels and suggests that CDCA7 functions as a transcriptional repressor.

### CDCA7 is required to maintain global DNA methylation levels in multiple tissues

**Fig. 2.**
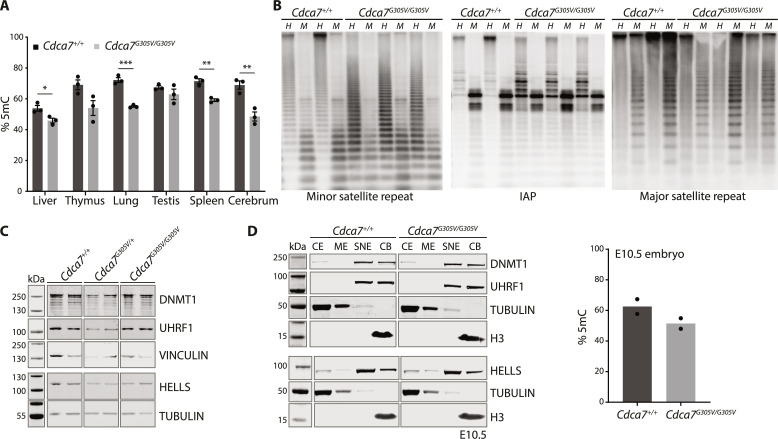
CDCA7 is required to maintain global DNA methylation levels in multiple tissues. (**A**) Bar chart showing DNA methylation levels in different tissues of P21 animals measured by LUMA. Three biological replicates per genotype [error bars = SEM; *P < 0.05, **P < 0.005, and ***P < 0.0005 (unpaired *t* test)]. (**B**) Southern blots showing minor satellite, intracisternal A particle (IAP), and major satellite repeat methylation levels in the P21 spleen. Genomic DNA was digested with *Hpa*II (*H*; methylation sensitive) or *Msp*I (*M*; methylation insensitive). Two or three biological replicates per genotype. (**C**) Western blot showing DNMT1, UHRF1, and HELLS protein levels in P21 spleen; two biological replicates per genotype. TUBULIN or VINCULIN were used as loading controls. (**D**) Left: Western blot showing DNMT1, HELLS, and UHRF1 protein levels in CE, ME, SNE, and CB isolated from E10.5 (four pooled embryos per genotype). TUBULIN and H3 were used as loading controls. Right: Bar chart showing DNA methylation levels in E10.5 embryos measured by LUMA; two biological replicates per genotype.

As aberrant genome-wide DNA hypomethylation has been found in the blood of patients with ICF carrying *CDCA7* missense mutations (*5*), we next used the luminometric methylation assay (LUMA), a method that relies on the presence of 5mCpG-sensitive restriction enzyme sites (*14*), to measure global methylation levels in P21 WT and *Cdca7^G305V^* homozygotes. We found significantly reduced global methylation levels in tissues from different germ layers in the mutants, with up to ~20% difference between WT and *Cdca7^G305V^* homozygotes (Fig. 2A).

Southern blot analysis revealed minor satellite repeat hypomethylation in *Cdca7^G305V^* mutant spleens when compared to WT. In addition, we found reduced methylation levels of intracisternal A particle (IAP) retrotransposons, while major satellite repeat methylation levels did not show major differences between WT and *Cdca7^G305V^* homozygotes (Fig. 2B). This pattern of repeat hypomethylation in *Cdca7^G305V^* mutants was found in both tissues with high (i.e., spleen and thymus) and low (i.e., liver) CDCA7 expression levels (Fig. 2B and fig. S3A). This effect on DNA methylation was not the result of the differential expression of key methylation machinery members in the spleen and thymus (Fig. 2C and fig. S3, B and C). Furthermore, cellular fractionation followed by Western blot in WT and *Cdca7^G305V^* homozygous embryos revealed no obvious differences in cellular localization of DNMT1, UHRF1, and LSH/HELLS using this bulk assay, while global DNA methylation levels were reduced in the homozygotes when measured by LUMA (Fig. 2D). In addition, minor satellite repeats and IAP elements showed hypomethylation in embryos, as observed in P21 *Cdca7^G305V^* mutant thymus and spleen (fig. S3D). Together, these results show that CDCA7 is critical to maintaining global DNA methylation levels across tissues and developmental stages in mice.

### CDCA7 primarily mediates DNA methylation of the B genomic compartment in the spleen

**Fig. 3.**
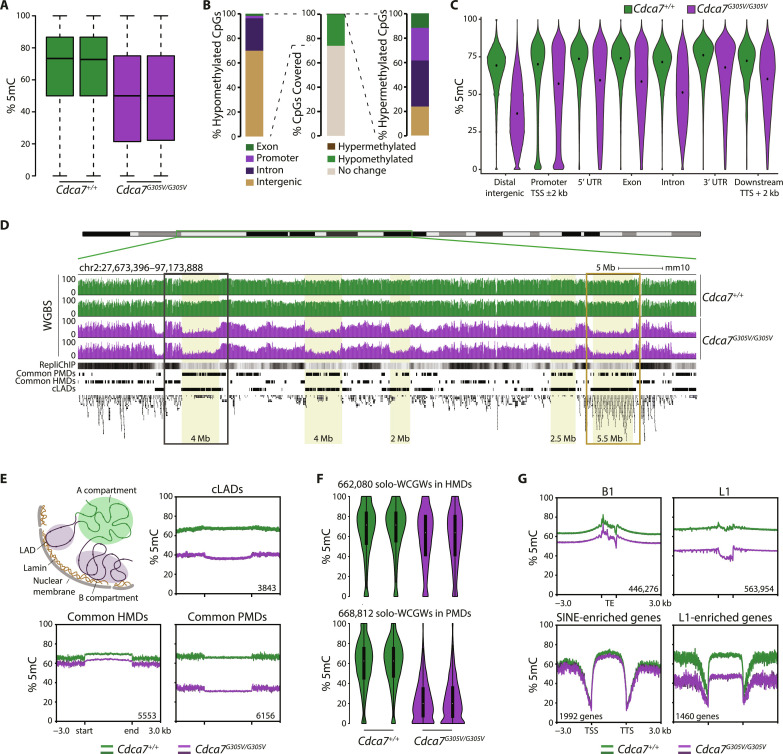
CDCA7 primarily mediates DNA methylation of the B genomic compartment in the spleen. (**A**) Boxplots slowing global methylation levels measured by WGBS. Each boxplot indicates one biological replicate [middle line indicates median, box limits indicate upper and lower quartiles, and whiskers extend to 1.5× interquartile range (IQR) from quartiles]. (**B**) Middle: Stacked bar chart showing proportions of differentially methylated or unchanged CpGs in *Cdca7^G305V^* homozygotes. Proportions of hypomethylated (left) or hypermethylated (right) CpG sites with respect to their genomic annotation. (**C**) Violin plots showing WGBS methylation levels at different genomic features (average methylation levels over 1-kb tiles). Average of two biological replicates per genotype. (**D**) Genome browser view depicting methylation profiles over part of chromosome 2; two replicates per genotype. Replication ChIP-seq data (ENCFF001JUT) and BED tracks for previously defined common partially methylated domains (PMDs) and highly methylated domains (HMDs) (*17*) and cLADs (GSE17051) and RefSeq annotations are shown. Yellow shading, representative hypomethylated domains; gray rectangle, representative gene poor hypomethylated region (zoom-in in fig. S4D, top); dark orange rectangle, representative hypomethylated gene cluster (zoom-in in fig. S4D, bottom). (**E**) Top: Active A (green) and inactive B (blue) genomic compartments of the nucleus are illustrated. Profile plots showing methylation levels ±3 kb over cLADs, (GSE17051). Bottom: Common HMDs and PMDs (*17*). Methylation levels were calculated over 50 bp for cLADs, and 10-bp bins for common PMDs and HMDs. Numbers indicate a number of regions over which average methylation was calculated. (**F**) Violin plots showing methylation at previously defined solo-WCGWs (*17*) located in common HMDs or PMDs; two biological replicates per genotype. The number indicates the number of CpGs plotted (fig. S5B). (**G**) Profile plots showing methylation levels ±3 kb over (top) L1 and B1 elements (calculated over 10-bp bins) and (bottom) over previously defined L1- and SINE-enriched genes (*21*) (calculated over 10-bp bins).

Next, to uncover the full extent of DNA methylation defects, we analyzed methylation levels genome-wide at base-pair resolution using WGBS on genomic DNA (gDNA) from two WT and two *Cdca7^G305V^* homozygous P21 spleens (fig. S4A). This revealed a global reduction in methylation in *Cdca7^G305V^* homozygotes (Fig. 3A), consistent with our LUMA results (Fig. 2A). The WGBS median methylation levels in *Cdca7^G305V/G305V^* and WT spleens were 51 and 73%, respectively (Fig. 3A). Using the following criteria: ≥15% methylation difference and *P* < 0.05, we found that 26.01% of all cytosines covered by WGBS (*n* = 19,199,377) were affected by the *Cdca7^G305V^* mutation (Fig. 3B). The vast majority of differentially methylated cytosines (99.98%) showed hypomethylation (Fig. 3B), with >75% losing between 30 and 60% methylation (fig. S4B). A total of 3609 cytosines (0.02%) gained DNA methylation (Fig. 3B). Although methylation changes affected all genomic elements analyzed, they were most pronounced in distal intergenic regions where average methylation levels dropped from ~67% in WT to ~38% in *Cdca7^G305V^* homozygotes (Fig. 3C).

A closer manual inspection and UCSC genome browser visualization indicated that methylation loss in *Cdca7^G305V^* homozygous spleens occurred over large domains and overlapped regions with predicted late replication timing, partial DNA methylation, and lamina-associated domains (LADs), which are found in many cell types throughout normal development (Fig. 3D and fig. S4D) (*15*–*19*). These observations therefore prompted us to examine whether CDCA7-associated methylation alterations affected genomic compartments rather than specific loci (Fig. 3E). Plotting methylation levels over previously defined constitutive LAD (cLAD) regions (*18*) revealed a ~30% methylation reduction in *Cdca7^G305V^* homozygotes. We found the same trend of reduced DNA methylation levels over common partially methylated domains (PMDs) that also overlap with cLADs (*17*) and genes embedded in PMDs (Fig. 3E and fig. S5A). On the other hand, the degree of methylation over highly methylated domains (HMDs) was only slightly affected (Fig. 3E). Next, we asked if previously identified solo-WCGW sites (*17*) are also susceptible to the *Cdca7^G305V^* mutation. Our analysis showed that the *Cdca7^G305V^* mutation had a greater impact on solo-WCGW located in PMDs compared to those found in HMDs. On average, solo-WCGW in PMDs lost ~40% methylation while at solo-WCGW in HMDs methylation was reduced by an average of ~5% (Fig. 3F and fig. S5B). It has been reported that Sine B1 (B1) and Line1 (L1) retrotransposons can be used to predict the A (active) and B (inactive) compartments of the genome, respectively (*20*). Plotting methylation levels over mouse B1 and L1 consensus sequences and ±3 kb of the surrounding regions revealed that methylation was more severely reduced (~30%) over L1 (average ~69% in WT versus ~39% in homozygotes) compared to B1 elements (~12%) (average ~72% in WT versus ~60% in homozygotes) in the mutants (Fig. 3G). Furthermore, using a previously reported list of B1- and L1-enriched genes (*21*), we found that L1-associated genes were more affected by methylation loss [average ~67% (WT) versus ~46% (mutants)] in *Cdca7^G305V^* homozygotes than B1-associated genes [average ~66% (WT) versus ~63% (mutants)] (Fig. 3G). Notably, analysis of 17 curated imprinted control regions (ICRs) revealed that they were not affected by *Cdca7^G305V^*-associated hypomethylation in the spleen (fig. S4C). Combined, these analyses show that CDCA7 preferably promotes methylation at partially methylated cytosines that reside in the B genomic compartment.

### Aberrant DNA hypomethylation is associated with the up-regulation of lowly expressed genes in the spleen of *Cdca7^G305V^* homozygotes

**Fig. 4.**
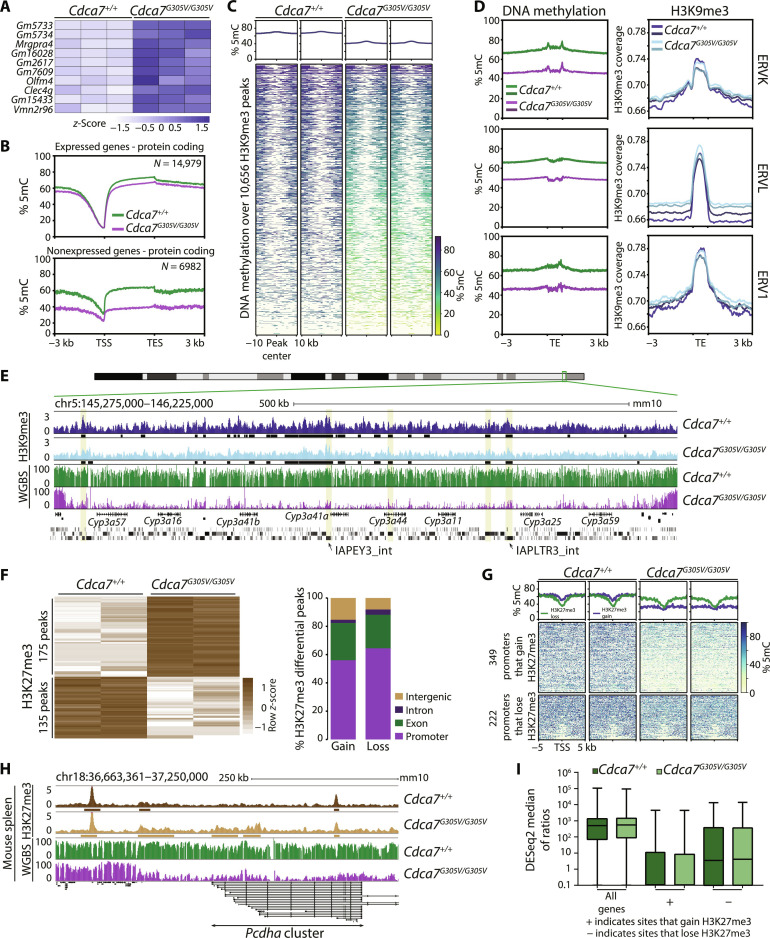
CDCA7 is dispensable for H3K9me3 deposition, while aberrant DNA hypomethylation is accompanied by increased H3K27me3 at gene clusters in the spleen. (**A**) Heatmap showing expression levels of the 10 differentially expressed genes. Light blue denotes low, and dark blue indicates high expression. (**B**) Profile plots depicting average methylation levels over protein-coding expressed and nonexpressed genes and 3-kb flanking regions in WT and *Cdca7^G305V^* homozygous spleen. (**C**) Profile plots and heatmaps showing CpG methylation over the center of H3K9me3 consensus peaks and flanking 10-kb regions (methylation levels calculated over 1-kb bins). (**D**) Profile plots showing (left) CpG methylation or (right) H3K9me3 levels at ERVK, ERVL, and ERV1 and 3-kb flanking regions in two WT and two *Cdca7^G305V^* homozygous P21 spleens (TE, transposable element). (**E**) Genome browser view depicting H3K9me3 and methylation over the *cytochrome P450* gene cluster. Black rectangles, H3K9me3 peaks; RefSeq and Repeat masker annotations are shown; Yellow shading, H3K9me3-covered IAP. (**F**) Left: Heatmap showing average H3K27me3 levels at 310 differentially enriched peaks in two WT and two *Cdca7^G305V^* homozygotes [DiffBind, default settings - DEseq2, false discovery rate (FDR) ≤ 0.05]. Right: Stacked bar chart showing the proportion of H3K27me3 differential peaks (gained or lost in *Cdca7^G305V^* homozygotes) with respect to genomic annotation. (**G**) DNA methylation levels over promoters (TSS ± 5 kb) that gained or lost H3K27me3 in *Cdca7^G305V^* homozygotes represented by profile plots and heatmaps (methylation levels calculated over 100-bp bins). (**H**) Genome browser screenshot of H3K27me3 and methylation levels over clustered *protocadherin alpha* genes. (**I**) Box plot showing median expression of all genes, and genes with promoter H3K27me3 gain or loss in *Cdca7^G305V^* homozygotes (DEseq2 median of ratios for each gene was used as a measure of expression; middle line indicates median, box limits indicate upper and lower quartiles, and whiskers extend to 1.5× IQR from quartiles).

Next, we carried out RNA-seq on WT and *Cdca7^G305V^* homozygous P21 spleens (*n* = 3 per genotype). Differential gene expression analysis identified 10 significantly differentially expressed genes (DEGs) between WT and *Cdca7^G305V^* mutants when using *P*adj ≤ 0.05 and log_2_FC ≥ 1.5 as cutoffs (Fig. 4A, fig. S6A, and data S1). All 10 genes were up-regulated and include two members of large gene clusters, *MAS-related GPR*, *member A4* (*Mrgpra4*), and *vomeronasal 2*, *receptor 96* (*Vmn2r96*), linked to pruriception (*22*) and pheromone reception (*23*), respectively, and poorly annotated protein-coding genes (*Gm2617* and *Gm5734* and a cluster comprising *Gm16028*, *Gm15433*, and *Gm7609*). In addition, mRNA levels of *C-type lectin domain family 4 member G* (*Clec4g*), which belongs to the C-type lectin-like receptors that play a role in the immune response (*24*), and *olfactomedin 4* (*Olfm4*), a member of the olfactomedin family that has roles in cell adhesion (*25*), were increased (Fig. 4A, fig. S6, A and B, and data S1).

We then compared gene expression with DNA methylation changes. Overall, transcriptionally silent genes were most affected by *Cdca7^G305V^*-associated hypomethylation (Fig. 4B), in agreement with our observation that CDCA7 preferably promotes DNA methylation of L1-enriched genes (Fig. 3G). Furthermore, the majority of mappable DEGs (*n* = 7/8) also covered by WGBS were located in regions that lost methylation (~30%) in *Cdca7^G305V^* mutants (fig. S6C). However, a closer manual inspection also revealed that six of eight DEGs already showed some low-level expression (threshold set to an average of ≥5 reads) in WT (fig. S6, B and D, and data S1). Thus, although we found that CDCA7 is necessary to maintain global DNA methylation levels in the spleen, the transcriptional responses to aberrant hypomethylation in *Cdca7^G305V^* homozygotes were largely confined to the up-regulation of a small set of lowly expressed genes. Notably, the silencing of TE elements was also not perturbed in the *Cdca7^G305V^* mutants in our bulk RNA-seq analysis (fig. S6E).

### CDCA7 promotes DNA methylation at H3K9me3 marked genomic sites but is dispensable for H3K9me3 deposition in spleen

The mild transcriptional effect despite the relatively large methylation differences observed in *Cdca7^G305V^* homozygotes suggests that silencing can be maintained by alternative mechanisms. The B genomic compartment is enriched for the repressive histone mark H3K9me3, and DNA methylation and H3K9me3 can be positively correlated (*2*). Therefore, we next investigated whether CDCA7 disruption also influenced H3K9me3 distribution and performed chromatin immunoprecipitation followed by high throughput sequencing (ChIP-seq) in the P21 spleen. We found that the majority of H3K9me3 peaks (72.3 and 72.8% out of *n* = 10,656 consensus peaks called for WT and *Cdca7^G305V^*, respectively) mapped to intergenic regions, where they often overlap with repetitive elements (fig. S7A). Intersecting WGBS and H3K9me3 ChIP-seq datasets revealed reduced DNA methylation at H3K9me3-enriched sites in *Cdca7^G305V^* mutant spleens (Fig. 4C). Notably, the effect of the *Cdca7^G305V^* mutation on deposition of H3K9me3 was minimal with <1% [*n* = 29; false discovery rate (FDR) ≤ 0.05] of peaks showing a differential enrichment between WT and *Cdca7^G305V^* spleens (fig. S7B and data S1). H3K9me3 enrichment in *Cdca7^G305V^* homozygous spleens was unchanged at known H3K9me3 sites including endogenous retroviruses (ERVs) (Fig. 4D and fig. S7C) and developmentally silenced genes (*26*) such as the *cytochrome P450* (Fig. 4E) and *zinc finger and SCAN domain containing 4* (*Zscan4*) gene clusters (fig. S7D), suggesting that reduced DNA methylation levels in *Cdca7^G305V^* mutants did not influence the repressive chromatin environment at these sites. Combined, these data suggest that CDCA7 is dispensable for the deposition of the heterochromatin mark H3K9me3 genome-wide and that the residual DNA methylation in combination with H3K9me3 is sufficient to maintain transcriptional repression of H3K9me3 target sites in *Cdca7^G305V^* mutant spleen.

### Aberrant DNA hypomethylation is accompanied by increased H3K27me3 at gene clusters in *Cdca7^G305V^* homozygous spleen

In addition to intergenic regions, promoters lost DNA methylation in *Cdca7^G305V^* spleens (Fig. 3C). While a negative correlation between promoter methylation and the repressive histone modification H3K27 trimethylation (H3K27me3) has been reported (*27*), it has also been shown that H3K27me3 distribution can be altered in DNA methylation mutants (*28*, *29*). Therefore, we next profiled H3K27me3 using ChIP-seq in WT and *Cdca7^G305V^* mutant P21 spleen (*n* = 2 per genotype). The majority of peaks (66 and 63%, respectively in WT and *Cdca7^G305V^* mutants) overlapped with promoters (fig. S8A). We identified a total of 310 H3K27me3 differentially enriched peaks (FDR ≤ 0.05) between WT and *Cdca7^G305V^* spleens. A total of 175 peaks were associated with H3K27me3 gain and 135 peaks were associated with H3K27me3 loss in the mutants, and, the differentially enriched peaks 52% (*n* = 92) and 63% (*n* = 85), respectively, were annotated as promoters [transcriptional start site (TSS) ± 1.5 kb] (Fig. 4F, fig. S8B, and data S1). A heatmap representation further revealed that the gain of H3K27me3 in *Cdca7^G305V^* mutants occurred at hypomethylated sites, whereas reduced H3K27me3 was independent of DNA methylation alterations (fig. S8C). We obtained a similar result when investigating promoter-associated (TSS ± 1.5 kb) peaks, which covered 571 promoters (Fig. 4G and data S1). Notably, around one-third of the peaks (34%, 59 of 175) where H3K27me3 gain is accompanied by methylation loss mapped to gene clusters and the transition to H3K27me3 extended over long (up to 217 kb) stretches, encompassing promoters and gene bodies. Notable examples include the clustered *protocadherin* (*Pcdh*) genes (Fig. 4H and fig. S9A), *olfactory receptor*, *keratin* cluster, and *vomeronasal receptor* genes (fig. S9B). Integrating gene expression and ChIP-seq data further showed that genes with H3K27me3 gain remained transcriptionally silent in *Cdca7^G305V^* mutants (Fig. 4I). From these data, we conclude that genome-wide H3K27me3 distribution is altered in *Cdca7^G305V^* mutant spleen and that higher enrichment of H3K27me3-associated factors at hypomethylated promoters could contribute to the sustained repression of the associated genes.

### Clustered *protocadherin* gene expression and chromatin state are dysregulated in *Cdca7^G305V^* homozygous embryonic brain

**Fig. 5.**
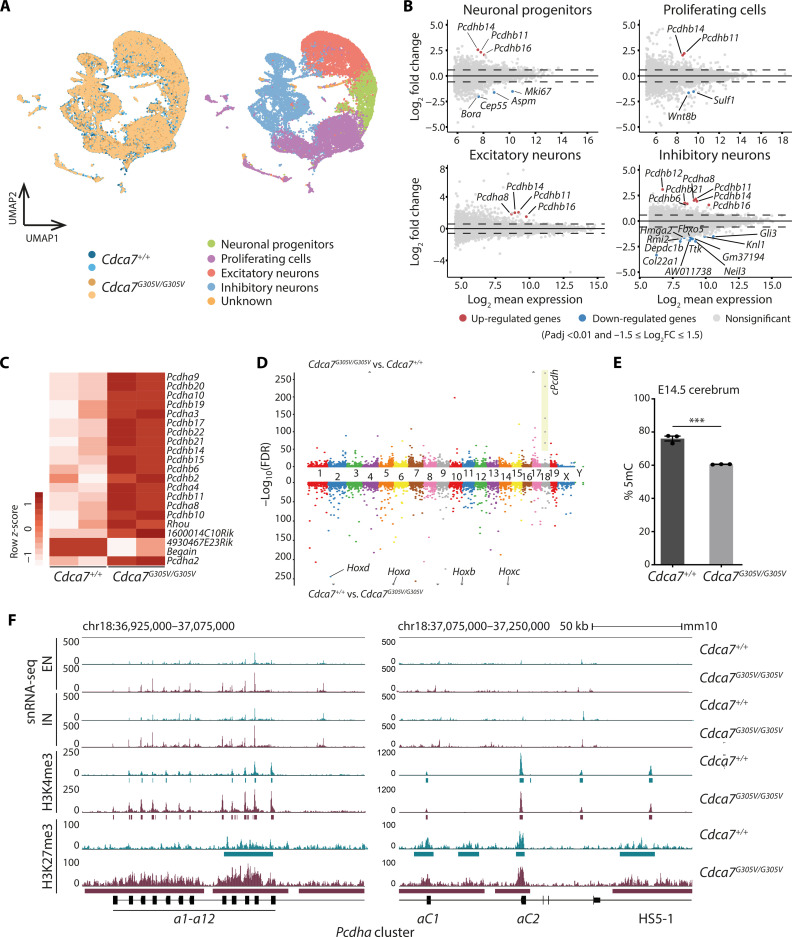
Dysregulation of clustered *protocadherin* gene expression and chromatin state in *Cdca7^G305V^* homozygous cerebrum. (**A**) Uniform Manifold Approximation and Projection (UMAP) representations of E14.5 cerebrum integrated snRNA-seq data (*n* = 25,923 nuclei). Left UMAP: The colors represent four different samples grouped by genotype. Right: Integrated dataset colored according to the four annotated cell types. A small number of nuclei (*n* = 201 in one WT and *n* = 104 in one *Cdca7^G305V/G305V^* biological replicate) that could not be assigned to a specific cell type were designated “unknown” and were not included in the subsequent analyses. (**B**) MA plots showing differential RNA levels between WT and *Cdca7^G305V^* homozygous E14.5 cerebrum for the different cell types. Red, significantly up-regulated; blue, significantly down-regulated genes (*P*adj ≤ 0.01 and −1.5 ≤ log_2_FC ≤ 1.5). Horizontal dashed lines represent log_2_FC thresholds of 1.5 and −1.5; *n* = 2 biological replicates per genotype. (**C**) Heatmap showing average H3K4me3 levels at 21 differentially enriched peaks in WT and *Cdca7^G305V^* homozygous E14.5 cerebrum (DiffBind, default settings - DEseq2, FDR ≤ 0.05). (**D**) Miami plot visualization of H3K27me3 ChIP-seq differential analysis using sicer_df showing (top) regions that gain H3K27me3 (*Cdca7^G305V^*/WT) and (bottom) regions that lose H3K27me3 (WT/*Cdca7^G305V^*). All top-scoring dots on chromosome 18 correspond to the clustered *Pcdh* locus (highlighted in yellow). All four *Hox* clusters lose H3K27me3 as indicated (triangles indicate out-of-the-range values). (**E**) Bar chart showing DNA methylation levels in E14.5 cerebrum measured by LUMA. Three biological replicates per genotype [error bar = SEM; ***P < 0.0005 (unpaired *t* test)]. (**F**) Genome browser screenshot of the clustered *Pcdha* locus. Representative tracks for snRNA-seq (EN, excitatory neurons; IN, inhibitory neurons) and ChIP-seq (H3K4me3 and H3K27me3) from E14.5 cerebrum are shown (rectangles below H3K4me3 and H3K27me3 tracks, called peaks; RefSeq annotation is shown below the tracks).

It has previously been reported that PMDs are enriched for gene clusters with brain-specific expression (*30*). Considering that we did not see large transcriptional deregulation in CDCA7 mutant spleens, we wondered if this was due to the fact that this tissue lacks transactivating factors required to take advantage of the aberrantly hypomethylated state. We therefore studied this in the E14.5 cerebrum where CDCA7 is expressed (fig. S10A), using droplet-based single-nucleus (sn) RNA-seq (*n* = 2 per genotype). After stringent filtering, a total of 25,923 nuclei were retained, then integrated and analyzed together (Fig. 5A). The expression of known neuronal marker genes (*31*) including a *marker of proliferation Ki-67* (*Mki67*) and *paired box 6* (*Pax6*) (proliferating cells), *eomesodermin* (*Eomes*) (neuronal progenitors), *glutamate decarboxylase 2* (*Gad2*) (inhibitory neurons), and *neuronal differentiation 6* (*Neurod6*) (excitatory neurons) was used to broadly annotate cell types (fig. S11, A and B), and *Cdca7* expression was detectable in all cell types in both genotypes (fig. S10, B to D). When averaged across all four samples, most cells (59%) were annotated as excitatory and inhibitory neurons. In addition, we detected proliferating cells (30%) and neuronal progenitors (9.6%) with high concordance between all four samples (fig. S11C). These results suggest that CDCA7 is dispensable for early neurogenesis, and we found no obvious phenotypic differences between WT and *Cdca7^G305V^* homozygous mutant brains (fig. S12A). Accordingly, pseudo-bulk transcriptome analyses (based on the snRNA-seq data) on the different cell populations identified only small numbers of DEGs (DESeq-2, Log_2_FC > 1.5, *P*adj < 0.01). Of note, proliferating cells and inhibitory neurons showed a mild, yet significant down-regulation of cell cycle and chromosome segregation genes, e.g., *abnormal spindle-like*, *microcephaly-associated* (*Aspm*), and *Centrosomal Protein 55* (*Cep55*) which have been linked to craniofacial development (*32*, *33*). All up-regulated genes in the *Cdca7^G305V^* cerebrum belonged to the clustered *Pcdh* genes and they showed increased expression in all cell populations (Fig. 5B and data S2).

We next investigated whether transcriptional dysregulation was reflected at the chromatin level using bulk-level native-ChIP (NChIP)-seq for the active histone mark H3K4me3 and the repressive modification H3K27me3, in E14.5 cerebrum. While the overall distribution of H3K4me3 was relatively unaffected by the *Cdca7^G305V^* mutation, we identified 21 differentially enriched peaks, 17 of which mapped to *Pcdha* isoforms and *Pcdhb* genes (Fig. 5C, fig. S12B, and data S1), in agreement with their increased expression observed by snRNA-seq (Fig. 5B). In addition, we found localized perturbation of H3K27me3 deposition in *Cdca7^G305V^* homozygous mutants. Genome-wide Miami plot visualization identified the c*Pcdh* and *homeobox* (*Hox*) gene clusters to be among the loci with the most significant H3K27me3 gain and loss in *Cdca7^G305V^* mutant cerebrum, respectively (Fig. 5D and fig. S12C), while the overall effect of CDCA7 disruption on H3K27me3 levels was modest and no difference in global H3K27me3 levels was observed (fig. S12D). Together, these data show that the transcriptional consequences of *Cdca7^G305V^*-associated ~16% global methylation loss in E14.5 cerebrum, as observed by LUMA (Fig. 5E), are largely confined to the *cPcdh* genes where they are accompanied by changes in chromatin state and gene expression (Fig. 5F and fig. S12E).

### CDCA7 is a modifier of clustered *protocadherin alpha* stochastic promoter choice

**Fig. 6.**
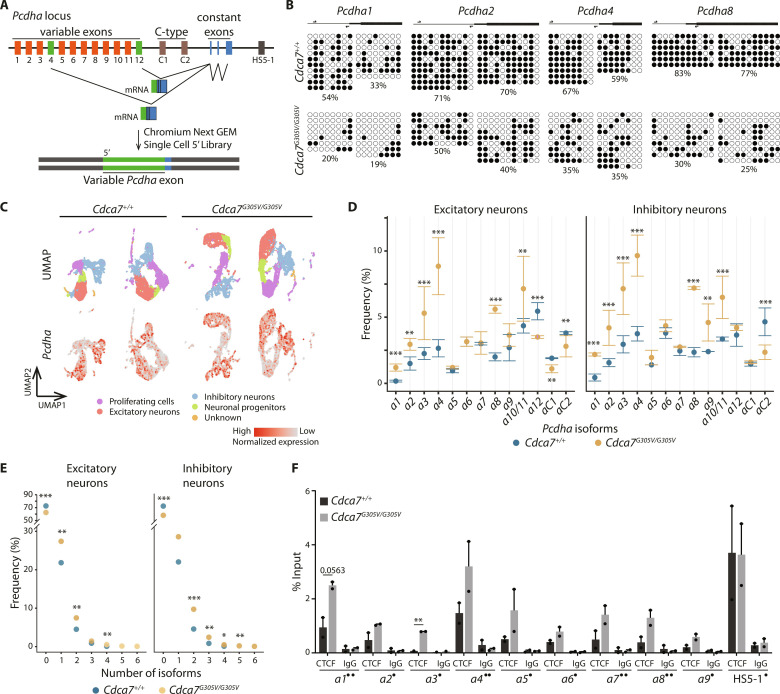
CDCA7 is a modifier of *protocadherin alpha* stochastic promoter choice. (**A**) Schematic of the *Pcdha* locus composed of variable and constant exons and experimental design. Each variable exon is spliced to the three constant exons. In a single neuron, one to five variable isoforms are randomly chosen and expressed (green rectangles). (**B**) Methylation levels of *Pcdha1*, *Pcdha5*, *Pcdha7*, and *Pcdha8* promoters were measured by bisulfite sequencing in WT and *Cdca7^G305V^* homozygous E14.5 cerebrum (filled circles, methylated cytosines; empty circles, unmethylated cytosines; black box, first exon; black arrows, primers). (**C**) Top UMAP of WT and *Cdca7^G305V^* homozygous E14.5 cerebrum (*n* = 2 per genotype). Single nuclei are colored by cell type. Bottom: Feature plots showing nuclei expressing *Pcdha* isoforms in the annotated cell types. (**D**) Quantification of *Pcdha* isoform expression frequencies in WT and *Cdca7^G305V^* homozygous excitatory and inhibitory neurons. Frequency (in percentage %) shown on the line graph is the mean ± standard error of the two biological replicates; normalized by the total number of nuclei for each cell type (fig. S11C). *Pcdha10/11* were combined because of difficulties with Cell Ranger annotation. Chi-square test *P < 0.05, **P < 0.01, and ***P < 0.001. (**E**) Quantification of the number of *Pcdha* isoforms expressed in WT and *Cdca7^G305V^* homozygous single nuclei in excitatory and inhibitory neurons. Frequency (in percentage %) shown is an average from two biological replicates per genotype; normalized by the total number of nuclei for each cell type (fig. S11C). Chi-square test *P < 0.05, **P < 0.01, and ***P < 0.001. (**F**) ChIP-qPCR showing CTCF occupancy at promoters of different variable *Pcdha* isoforms and the HS5-1 enhancer; two biological replicates per genotype. One dot indicates primers spanning conserved sequence element (CSE) before exon 1; two dots indicate primers spanning exonic CSE (eCSE). **P < 0.005 (two-tailed unpaired *t* test; error bars = SEM).

In the mouse, the *Pcdha* gene cluster consists of 12 variables and 2 constitutive isoforms (Fig. 6A), and combinatorial expression of individual variable isoforms in single neurons has been suggested to generate a barcode critical for neuronal diversity and network formation (*34*). Previous studies have estimated that, depending on neuronal type and developmental stage, in individual neurons between one and five variable *Pcdha* isoforms are expressed (*35*–*40*), likely the result of stochastic epigenetic processes in cells of the same type.

At the molecular level, both H3K9me3 (*41*, *42*) and DNA methylation pathways (*35*, *43*) have been implicated in the regulation of *Pcdha* expression and stochastic isoform choice. Using Sanger bisulfite sequencing, we found reduced *Pcdha* promoter DNA methylation levels in *Cdca7^G305V^* mutant cerebrum at all variable isoforms tested (Fig. 6B and fig. S13A), consistent with a role for CDCA7 in DNA methylation regulation. We also considered the effect of the *Cdca7^G305V^* substitution at the R1 site, where SET domain bifurcated 1 (Setdb1)–mediated H3K9me3 has previously been shown to influence *cPcdh* expression (*41*). We observed no substantial differences in H3K9me3 levels between WT and *Cdca7^G305V^* mutants at the R1 site when using ChIP-qPCR (fig. S13B) or at global levels (fig. S13C). This suggests that this site is epigenetically controlled by a pathway that does not require CDCA7 and is in agreement with our finding that CDCA7 disruption does not affect H3K9me3 deposition in the spleen. However, profiling of the entire *Pcdh* locus or genome-wide will be required to exclude *Cdca7^G305V^*-associated changes in H3K9me3 enrichment elsewhere.

Next, we examined if the altered *Pcdha* chromatin environment influenced the mechanism of variable isoform choice in the *Cdca7^G305V^* homozygotes. Since our snRNA-seq experiment was carried out using the Chromium 5′-library, this allowed us to analyze expression frequencies of variable *Pcdha* isoforms in the different cell types because we can distinguish sequencing reads from most, except *Pcdha10/11*, individual *Pcdha* cluster members (Fig. 6A). We detected nuclei expressing *Pcdha* isoforms in all annotated cell types including neuronal progenitors in both WT and *Cdca7^G305V^* mutants (Fig. 6C). In WT, we found that isoforms positioned at the 5′ end of the gene cluster (i.e., *Pcdha1* and *a2*) showed an overall lower expression frequency than isoforms located at the 3′ end (i.e., *Pcdha12*) (fig. S14A). In addition, we noticed that the *Pcdha3*, *-4*, and -*6* isoforms were expressed at a higher frequency (fig. S14A), which could suggest their favored use in the E14.5 cerebrum. Heatmaps revealed that this pattern of *Pcdha* isoform expression was disrupted in the *Cdca7^G305V^* cerebrum in all cell types analyzed (fig. S14B). The rarely used *Pcdha1*, *a8*, and several additional variable isoforms became expressed at significantly higher frequencies in the mutants (Fig. 6D, fig. S14, B and C, and data S2), concomitant with DNA methylation loss (Fig. 6B) and a gain in H3K4me3 (Fig. 5F). This pattern of increased expression likelihood was also reflected at the single cell level. We observed significantly higher numbers of nuclei expressing *Pcdha* isoforms and increased numbers of isoforms per individual neuronal cell in *Cdca7^G305V^* homozygotes (up to *n* = 6) when compared to WT (up to *n* = 4), in all assigned cell types (Fig. 6E, fig. S14D, and data S2). We then measured occupancy of the transcriptional regulator CTCF at variable *Pcdha* isoforms, since *Pcdha* promoters and the HS5-1 enhancer contain CTCF motifs (*44*–*46*). ChIP-qPCR experiments revealed increased CTCF binding in *Cdca7^G305V^* mutant cerebrum at variable *Pcdha* isoforms (Fig. 6F) that also showed increased expression frequency (Fig. 6D) and DNA hypomethylation (Fig. 6B and fig. S13A). This is consistent with previous reports that CTCF binding directly correlates with *Pcdha* isoform expression (*43*, *44*, *46*).

**Fig. 7.**
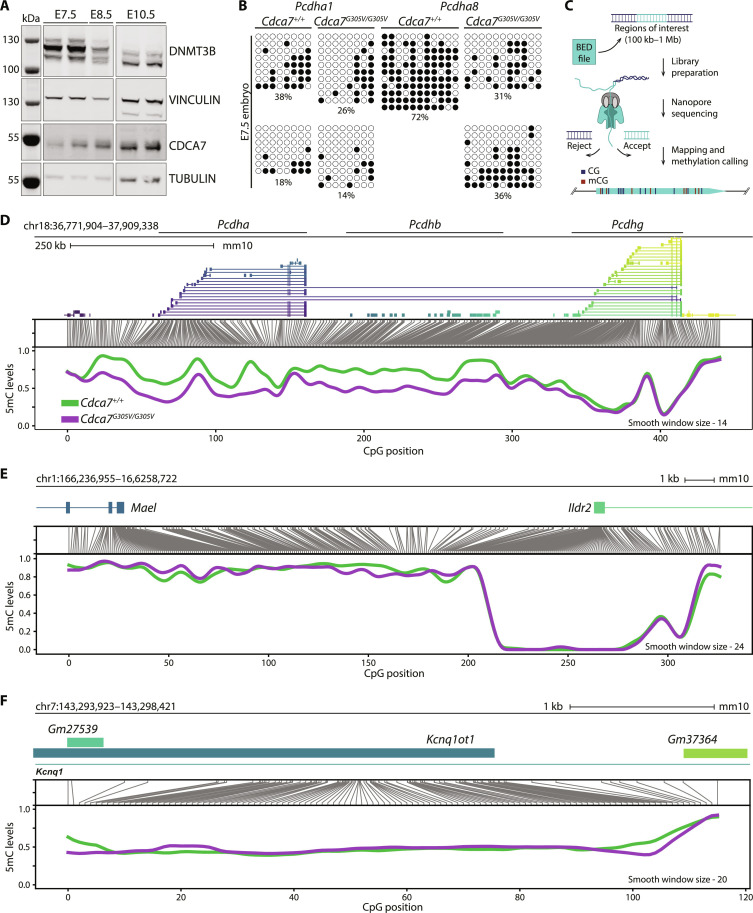
CDCA7 is required for *Pcdh* cluster methylation in the embryo. (**A**) Western blot showing DNMT3B and CDCA7 protein levels in E7.5, E8.5, and E10.5 whole WT embryos; VINCULIN and TUBULIN were used as loading controls. (**B**) DNA methylation levels of *Pcdha1* and *Pcdha8* promoters measured by Sanger bisulfite sequencing in WT and *Cdca7^G305V^* homozygous E7.5 whole embryos (filled circles, methylated cytosines; empty circle, unmethylated cytosines). (**C**) Schematic representation of pipeline for adaptive sampling with Oxford Nanopore Technology, where only regions of interest, provided within a BED file, are sequenced. (**D**) Methylation levels over the *Pcdh* cluster, (**E**) the *Mael* and *IIdr2* promoter regions, and (**F**) the *Kcnq1* imprinted region obtained from ONT long-read sequencing. From top to bottom, the figure panels show (i) the genomic position of interest, (ii) a diagram displaying the correspondence between genome space and CpG space, and (iii) the smoothed methylated fraction plot.

*Pcdha* promoter DNA methylation is acquired in the developing embryo (*35*, *47*) and relies on DNMT3B activity (*35*, *48*). Recessive mutations in *CDCA7* or *DNMT3B* can cause ICF syndrome (*3*, *49*–*52*). Therefore, we considered a potential connection between the two factors. By Western blot, we detected both CDCA7 and DNMT3B protein in E7.5 to E10.5 single embryos (Fig. 7A). We then measured DNA methylation levels of the *Pcdha1* and *Pcdha8* promoters by Sanger bisulfite sequencing and found reduced methylation in the *Cdca7^G305V^* mutants at E7.5 (Fig. 7B). To corroborate a potential *Pcdh* cluster DNA methylation defect in *Cdca7^G305V^* mutants, we performed an adaptive sampling target enrichment experiment using Oxford Nanopore Technologies (ONT) long-read sequencing (Fig. 7C). We focused on investigating DNA methylation of the *Pcdh* cluster genes and included representative germline genes [*maelstrom spermatogenic transposon silencer* (*Mael*) and *ribosomal protein L10 like* (*Rpl10l*)], imprinted genes [*potassium voltage-gated channel subfamily q member 1* (*Kcnq1*) and *paternally expressed 13* (*Peg13*)], and *Cdca7* as control loci (Fig. 7, D to F, and figs. S15 to S20). We observed reduced methylation over the *Pcdha* and *Pcdhb* genes, while *Pcdhg* members did not seem to be affected by the *Cdca7^G305V^* mutation (Fig. 7D and figs. S15 to S17), consistent with our findings in the spleen (Fig. 4H and fig. S9A). In agreement with results from patients with CDCA7/ICF3 (*5*), germline gene promoter methylation was not impaired in the mutant embryo (Fig. 7E and fig. S18). Furthermore, we found that methylation of DNMT1-dependent (*53*) germline differentially methylated region (DMR) of the two imprinted loci tested was preserved in the *Cdca7^G305V^* homozygote (Fig. 7F and fig. S19). These results show that CDCA7-associated aberrant c*Pcdh* gene DNA hypomethylation is present during a developmental window when DNMT3B is expressed. However, since DNMT1 is required to maintain DNA methylation at E8.5 genome-wide (*53*), further investigation will be necessary to determine how the DNMTs and CDCA7 are connected. Combined, our in vivo data show that global aberrant DNA hypomethylation can translate to localized changes in chromatin state and gene expression and suggest that CDCA7 promotes DNA methylation at gene clusters where this epigenetic mark is used as a modulator of expression likelihood.

## DISCUSSION

**Fig. 8.**
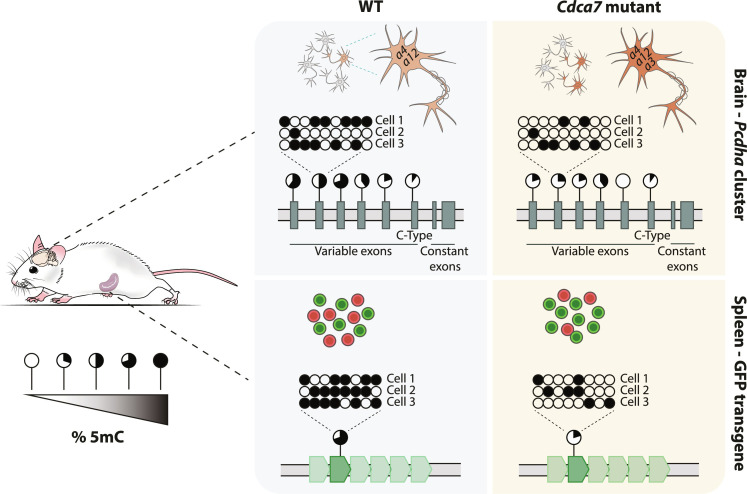
Model of CDCA7-mediated DNA methylation utilization at two stochastically expressed loci. The multicopy GFP transgene and the variable *Pcdha* promoters are characterized by mosaic DNA methylation, which is associated with variegated transgene expression in erythrocytes and influences stochastic *Pcdha* isoform choice in neuronal cells, respectively. CDCA7 disruption leads to hypomethylation and an increase in the probability of a cell expressing GFP in erythrocytes and *Pcdha* isoforms in neurons.

The number of reports where genome-wide defects in DNA methylation patterning are observed in individuals with germline mutations in epigenetic regulators is increasing. An important question that needs to be addressed when such widespread aberrant DNA methylation patterns are observed in developmental disease is how methylation alterations translate to gene expression changes. ICF syndrome is an exemplar disorder where abnormal DNA hypomethylation has been suggested to underlie disease phenotypic aspects (*54*). Using a mouse model carrying a germline ICF3/CDCA7 syndrome pathogenic variant, we show that CDCA7 is critical to maintaining global DNA methylation levels across multiple tissues and find that it functions as a transcriptional repressor by promoting DNA methylation of predominantly the B genomic compartment. CDCA7 disruption leads to the formation of large aberrantly hypomethylated domains, without affecting the deposition of the heterochromatin mark H3K9me3 in the spleen. Notably, ICF3 pathogenic CDCA7-associated aberrant hypomethylation translated to tissue-specific transcriptional dysregulation that affected distinct sets of genes. Deregulation correlated with the activity of the locus, rather than aberrant hypomethylation per se, thereby providing an intriguing example of how global DNA methylation alterations can result in localized, tissue-specific gene expression changes (Fig. 8). However, a current limitation of this model and our study is that DNA methylation levels were profiled in bulk, in tissues with mixed cell types. Therefore, we currently cannot exclude that partial methylation loss is a result of cellular heterogeneity.

Homozygous CDCA7 disruption does not affect the viability of mice up to 3 weeks of age. This phenotype was unexpected and makes CDCA7 stand out from other ICF-relevant HELLS/LSH (*55*) or DNMT3B knockout mouse models (*51*), which result in perinatal or embryonic lethality. Perhaps the closest resemblance can be seen between *Cdca7* and two previously described hypomorphic *Dnmt3b* mouse models (*13*, *56*), as well as hypomorphic HELLS and DNMT1 mouse models (*57*, *58*), where at least partial homozygous viability to adulthood has been reported. Possible explanations for *Cdca7^G305V^* homozygous viability may be related to the global methylation reduction of ~15% in the tissues tested, which is lower than what is, for instance, observed in embryonic lethal DNMT1 (*59*) or UHRF1 (*60*) knockout models. Furthermore, the characteristics of the aberrantly hypomethylated regions (located in the partially methylated, gene-poor, transcriptionally silent B genomic compartment) (Fig. 3, D and G) and the mild, tissue-specific, disruption of gene expression patterns that we observed in the P21 spleen and E14.5 cerebrum (Figs. 4A and 5B) could explain viability. There may also be functional redundancy between CDCA7 and its paralog cell division cycle associated 7–like (CDCA7L) at some genomic sites since we have implicated both factors in DNA methylation regulation in humans (*8*). Notably, PMDs are a major target of aging-related DNA methylation alterations (*17*, *61*), and premature aging has been show to occur in HELLS hypomorphic mutants (*57*). Therefore, a more extensive phenotyping of the *Cdca7^G305V^* line will be required to reveal the full extent of transcriptional dysregulation and any phenotypes that may emerge during adulthood.

Our protein subcellular fractionation experiments revealed that CDCA7 is mainly bound to chromatin in vivo. The introduction of the ICF3 pathogenic G305V substitution largely abolished its chromatin localization (Fig. 1E). This is in agreement with published in vitro data, using frog-specific embryonic CDCA7 (CDCA7e) from *Xenopus* egg extract and a different set of ZF-located ICF3 missense mutations (*9*). The same study reported that missense mutations in the ZF-domain of CDCA7e lead to reduced association of HELLS with chromatin in vitro. However, in vivo, we observed no visible reduction of HELLS protein in the chromatin-bound fraction in *Cdca7^G305V^* homozygous mutants (Fig. 2D). Possible explanations for the observed differences could be that different experimental systems were used, in vitro versus in vivo or that our current assay is not sensitive enough. We also found that two other key factors of the maintenance machinery, UHRF1 and DNMT1, remain detectable in the chromatin-bound fraction in *Cdca7^G305V^* mutant embryos, at amounts comparable to WT (Fig. 2D). While UHRF1 and DNMT1 are responsible for DNA methylation maintenance genome-wide (*2*), our study points to a specific link between CDCA7 and DNA methylation maintenance of the B genomic compartment that overlaps with heterochromatic, late-replicating regions. At these sites, DNA methylation is markedly reduced in *Cdca7^G305V^* homozygotes (Fig. 3). It is therefore possible that only a fraction of cellular UHRF1 and DNMT1 are associated with CDCA7.

A recent evolutionary analysis could trace back CDCA7 and HELLS to the last eukaryotic common ancestor (*62*), suggesting conserved functions in DNA methylation pathways. We find that CDCA7 predominantly targets the B genomic compartment (Fig. 3, E and G), where its function seems to converge with HELLS (*63*). How this specificity is achieved remains to be determined. *Xenopus* CDCA7e contains a CXXC-ZF domain that can bind DNA in vitro (*9*). One possibility could be that CDCA7 occupies these sites via direct DNA binding. It is also possible that CDCA7 engages in protein-protein interactions to promote DNA methylation. At late-replicating regions, DNA methylation is maintained by replication-uncoupled maintenance (*64*). In human cell lines, this process has been shown to rely on the interaction of the UHRF1 tandem tudor domain with methylated H3K9 and the chromatin remodeling activity of HELLS/LSH (*65*), and HELLS/LSH enhances UHRF1 chromatin association (*66*). Therefore, CDCA7 could participate in replication-uncoupled methylation maintenance through its interaction with HELLS/LSH (*7*, *9*, *64*). However, the exact mechanisms of how CDCA7 connects with its target sites and participates in the maintenance of DNA methylation remain to be determined.

We provide base-pair resolution DNA methylation maps for CDCA7 and show that CDCA7 promotes methylation beyond the previously described minor satellite repeats in mice (Fig. 3C). We find that CDCA7 influences methylation levels of cytosines located at H3K9me3-decorated sites (Fig. 4C), in agreement with previous observations in humans (*8*), and suggesting that this role is conserved between species. We found that the heterochromatic mark H3K9me3 was maintained at CDCA7 aberrantly hypomethylated regions (Fig. 4E) and ERV superfamily transposable elements (TEs) in the spleen (Fig. 4D and fig. S7C). TEs remained transcriptionally silent (fig. S6E). In this respect, CDCA7 differs from previously reported HELLS mouse mutant cell lines, where reduced levels of both marks have been observed and linked to transcriptional dysregulation of ERV superfamily TEs (*28*, *63*). Currently, we do not know the mechanisms underlying these differences and whether they are specific to certain tissues or cell types. It could be that HELLS can associate with the H3K9me3 pathway independently of CDCA7 and that loss of both marks is required for TE derepression. At the same time, we cannot rule out differences in the severity of CDCA7 and HELLS hypomethylation defects at TEs and possibly other genomic sites in the mouse, which could influence H3K9me3 levels and transcriptional responses to DNA methylation loss.

We observed an altered H3K27me3 distribution over promoters and gene bodies in *Cdca7^G305V^* mutant spleen and cerebrum (Fig. 4F and fig. S12D), where H3K27me3 gain correlated with aberrant hypomethylation (Fig. 4G). These results are consistent with previous observations in DNMT1 (*29*), HELLS (*28*, *63*), and UHRF1 (*67*) mutant mouse models, where H3K27me3 gain at hypomethylated regions and loss of H3K27me3 at prominent Polycomb target genes such as the *Hox* clusters have been described. However, unlike the DNMT1 and UHRF1 models (*29*, *67*), we do not observe the up-regulation of *Hox* cluster gene expression in *Cdca7^G305V^* mutants (Figs. 4A and 5B). This could be explained by the more subtle DNA methylation and H3K27me3 changes observed in *Cdca7^G305V^* mutants. H3K27me3 gain did not occur at all aberrantly hypomethylated sites in the spleen, and we also did not identify any obvious features at genomic sites where H3K27me3 was lost in the *Cdca7^G305V^* mutants. This suggests that the distribution of these marks relies on a multilayered regulation, in line with a previous report (*29*).

Our study identifies the clustered *protocadherin* genes as prominent targets of ICF3 pathogenic CDCA7-associated aberrant DNA hypomethylation in the spleen and cerebrum (Figs. 4H and 6B). Notably, we observe transcriptional dysregulation exclusively in the *Cdca7^G305V^* mutant cerebrum (Figs. 4A and 5B), where this locus is normally active (*36*). This suggests that methylation loss per se is not sufficient to support transcription. Likely, the activity of *Pcdh* gene cluster members needs to be tightly controlled by several layers of epigenetic regulation to achieve cell type–specific stochastic expression. Consistent with this hypothesis are previous studies by others and us, which identified the *cPcdh* genes as targets of the de novo DNA methyltransferase DNMT3B (*35*), the human silencing hub (HUSH) complex (*41*, *42*) and the cohesion/CTCF complex member widely interspaced ZF motifs (WIZ) (*68*, *69*) via in vivo genetic approaches. Furthermore, CTCF is critical for *cPcdh* regulation by mediating promoter-enhancer interactions (*43*, *70*). In neuronal cells, we find increased CTCF binding at aberrantly hypomethylated *Pcdha* promoters, which correlated with increased *Pcdha* isoform expression in *Cdca7^G305V^* mutants (Fig. 6). These results are consistent with a role for CTCF in regulation of clustered *protocadherin* gene expression (*45*, *71*) and with previous reports of an inverse relationship between DNA methylation and reduced CTCF binding at imprinted genes (*72*, *73*), variably methylated IAP elements (*74*), and the *Pcdha* cluster (*41*). Besides, we observed an increase in the proportion of neuronal cells expressing *Pcdha* isoforms and individual neuronal cells expressed a higher number of variable isoforms (Fig. 6, D and E). Cell-to-cell variation in *Pcdha* DNA methylation levels and CTCF binding could potentially explain how higher numbers of *Pcdha* isoforms become activated in individual neuronal cells in *Cdca7^G305V^* mutants. In sum, although it remains to be determined whether individual *Cdca7^G305V^* mutant neurons exhibit changes in *Pcdha* promoter-enhancer interactions and we cannot exclude additional roles for CDCA7, it seems possible that DNA methylation is used to limit CTCF binding to specific *Pcdha* members and thereby safeguards isoform diversity (stochastic choice) in individual neurons.

While of the three *Pcdh* gene clusters, *Pcdha* and *Pcdhb* follow a similar pattern of dysregulation (aberrant hypomethylation and H3K27me3 gain in the spleen and H3K27me3 gain and isoform choice dysregulation in cerebrum) in the *Cdca7^G305V^* mutants, the *Pcdhg* cluster appears hardly affected at both the epigenetic and transcriptional level (figs. S9A and S12E). In addition to CDCA7, the disruption of WIZ (only the *Pcdhb* genes are dysregulated) or the structural maintenance of chromosomes flexible hinge domain containing 1 (SMCHD1) (up-regulation of *Pcdha* and *Pcdhb* cluster members but hardly any effect on the *Pcdhg* cluster) differentially affects the regulation of the *cPcdh* genes (*68*, *75*). Furthermore, recent work investigating histone modification dynamics at the *cPcdh* genes during mouse embryonic brain development found differences in H3K27me3 deposition (*76*). While H3K27me3 is present over the *Pcdhg* cluster from E10.5-P0, this mark appears to be lost from the *Pcdha* cluster around E13.5 (*76*). Together, this suggests that although the *Pcdha* and *Pcdhg* clusters share a similar genomic organization (*77*), their epigenetic regulation differs, as we observe in the *Cdca7^G305V^* mutants.

ICF syndrome is a genetically heterogeneous disease and can be caused by mutations in *DNMT3B *(*49*, *51*, *52*), ZF and BTB domain containing 24 (*ZBTB24*) (*78*, *79*), *CDCA7* (*3*), *HELLS* (*3*), or *UHRF1* (*80*). Our data suggest that CDCA7 functionally overlaps in the regulation of clustered *Pcdh* gene expression with at least two other ICF syndrome–causing factors, the de novo DNA methyltransferase DNMT3B and the chromatin remodeler HELLS, such that their disruption leads to aberrant hypomethylation and increased expression of *Pcdh* isoforms in neuronal tissue (*35*) or mouse embryonic fibroblasts (MEFs) (*63*), respectively. This is intriguing since the *PCDHA* promoters are among the few loci found to be aberrantly hypomethylated in the blood of patients with ICF carrying *DNMT3B*, *ZBTB24*, *CDCA7*, *HELLS*, or *UHRF1* mutations (*5*, *80*). Considering that the clustered *PCDH* genes are critical for generating neuronal diversity (*81*, *82*) and their suggested links to neurological disease (*83*, *84*), clustered *PCDH* dysregulation could be a potential contributor to the neurodevelopmental symptoms including intellectual disability that have been described in patients with ICF (*3*).

## MATERIALS AND METHODS

### Ethics statement

All procedures involving animals were approved by the Animal Ethics Committee of the Leiden University Medical Center and by the Commission of Biotechnology in Animals of the Dutch Ministry of Agriculture.

### *Cdca7^G305V^* mouse model

The *Cdca7^G305V^* mouse model was generated using CRISPR-Cas9 technology and pronuclear injection of E0.5 oocytes (FVB/NJ Line3 background) with the following mixture: Cas9 mRNA (25 ng/μl), *Cdca7* gRNA mRNA (12.5 ng/μl), ssODN [151 nucleotides (nt) carrying the desired point mutation; 15 ng/μl 5′- catgtcatcagtgtcgccagaaaaccactgacaccaaaaccaactgccgaaacccagactgctggggcatccggg**T**cca**g**ttctgtggtccctgccttcgaaaccgctatggcgaggaggtcaaggacgctctgctggatccggtaagtgg - 3′]. *Cdca7* gRNA 3′-AGGGACCACAGAATTGGCCC(CGG) - 5′ targets (anti-sense) exon 7 of mouse *Cdca7* (chr2:72,315,069 to 72,315,088). The CRISPR was designed with http://crispor.tefor.net/ and showed four potential off-target sites (fig. S1A and table S1). The *Cdca7^G305V^* line was maintained as heterozygotes. All experimental data were obtained using heterozygous *Cdca7^G305V^* mice five generations or more removed from the founders.

### Embryo dissections

All embryos were produced by natural timed matings. Noon of the day that the vaginal plug was detected was considered E0.5.

### DNA isolation

gDNA was isolated using the salt-extraction method. Briefly, tissues were lysed in cell lysis buffer [50 mM tris-HCl (pH 8), 4 mM EDTA (pH 8), and 2% SDS] plus proteinase K (390973P, VWR) and incubated at 55°C overnight. Cell lysate was treated with ribonuclease A (EN0531, Thermo Fisher Scientific) at 37°C for 1 hour. Saturated NaCl buffer was added, followed by the addition of isopropanol to precipitate gDNA and washing with 70% ethanol (EtOH). gDNA was dissolved in water and concentration was measured using NanoDrop.

### Genotyping

gDNA was used as a template for genotyping using DreamTaq Polymerase (EP0705, Thermo Fisher Scientific) and the following primers: 5′- AGGCCTTTTTGACCAGTGTG - 3′ (forward) and 5′- GCATCACACTGTGGTCTCCT – 3′ (reverse). PCR products were purified with Exonuclease I (70073X, 5000 UN, Affymetrix) and FastAp Thermosensitive Alkaline Phosphatase (EF0654, Thermo Fisher Scientific) for 1 hour at 37°C and 15 min at 80°C before Sanger sequencing.

### RNA isolation and RT-qPCR

Total RNA was isolated with QIAzol (5346994, Qiagen). About 1 μg of total RNA was used for reverse transcription using RevertAid H Minus First Strand cDNA Synthesis Kit (K1632, Thermo Fisher Scientific). RT-qPCR was performed in triplicate on a C1000TM Thermal cycler (Bio-Rad) with SYBR Green (170-8887, Bio-Rad). Expression data were normalized to β-*actin* and CFX manager (Bio-Rad) was used for data analyses and GraphPad for visualization. Primer sequences are provided in table S1.

### Protein isolation

Tissues were lysed in radioimmunoprecipitation assay (RIPA) buffer (0.1% SDS, 1% NP-40, 150 mM NaCl, 5 mM EDTA, 0.5% sodium deoxycholate, 20 mM tris-HCl) with 1× protease inhibitor cocktail (27368400, Roche) on ice using glass beads (0.5 mm, GB05, Bio-Connect), and Bullet Blender Storm 24 (Next Advance, intensity 10, 3 min). BCA kit (23225, Thermo Fisher Scientific) was used to measure protein concentration.

### Subcellular protein fractionation

Subcellular protein fractionation was performed according to the manufacturer’s instructions (87790, Thermo Fisher Scientific). Briefly, frozen tissues, stored at −80 after dissection, were pooled by genotype and homogenized using a Dounce homogenizer (12 strokes with A pestle followed by 6 to 12 strokes with B pestle). E10.5 tissue was further processed for 50 mg weight according to the protocol (87790, Thermo Fisher Scientific). A BCA kit (23225, Thermo Fisher Scientific) was used to measure the protein concentration of each fraction.

### Western blot

Total cell extracts or different cellular fractions were loaded on a NuPAGE gel (4 to 12%, NP0321, Thermo Fisher Scientific) and transferred to a Nitrocellulose Blotting Membrane (10600016, Life Sciences). The following primary antibodies were used: CDCA7 (1:500; 15249-1-AP, Proteintech), DNMT3A (1:1000; ab13888, Abcam), DNMT3B (1:1000; ab16049, Abcam), HELLS (1:1000; 11955-1-AP, Proteintech), UHRF1 (1:1000; 12387, Cell Signaling Technology), DNMT1 (1:1000; 5032, Cell Signaling Technology), Tubulin (1:2000; T6199, Sigma-Aldrich), Vinculin (1:2000; V9131, Merck), and H3 (96C10, 3638, Cell Signaling Technology). Donkey anti-Rabbit 800CW (1:5000; 926-32213, Li-Cor), Donkey anti-mouse 680RD (1:5000; 926-68072, Li-Cor), Donkey anti-Mouse 800CW (1:5000; 926-32212, Li-Cor), and Goat anti-Rabbit 680RD (1:5000; 926-68071, Li-Cor) were used as secondary antibodies. Membranes were analyzed using Odyssey (Westburg).

### Luminometric methylation assay

For each sample, 1200 ng of gDNA was digested with 5 U each of HpaII (ER0512, Thermo Fisher Scientific) or MspI (ER0542, Thermo Fisher Scientific) in an end volume of 10 μl in 2× TangoY buffer (Thermo Fisher Scientific) for 4 hours at 37°C. After digestion, 5.5 μl of the sample was loaded on a PyroMark Q48 Autoprep machine (9002471, Qiagen) using PyroMark Q48 Advanced reagents (974002, Qiagen). The following nucleotide dispensation order was used: GTGTCACATGTGTG (*85*). The formula 1-[(HpaII(G)/EcoRI(T)/(MspI(G)/EcoRI(T))] × 100, where G and T represent nucleotides #9 and #8, respectively, was used to calculate % global methylation from the obtained pyrograms.

### Sanger bisulfite sequencing

One microgram of gDNA was bisulfite converted using the Zymo Lightning Kit (D5030, BaseClear) according to the manufacturer’s instructions. PCR products were amplified using HotStarTaq DNA polymerase (87722, Thermo Fisher Scientific), cloned into the TOPO TA cloning kit in pCR2.1 (450641, Thermo Fisher Scientific), and transformed into *Escherichia coli* DH5α. DNA from individual colonies was Sanger-sequenced. Sequences with a conversion rate of at least 96% were analyzed using BiQ Analyzer software (*86*). Lollipop figures were made using the Quantification tool for Methylation Analysis (QUMA) (*87*). Primer sequences are provided in table S1.

### Southern blot

Southern blotting was used to assess DNA methylation levels at satellite repeats and IAPs. Briefly, 1 μg of gDNA was digested with *Hpa*II (methylation sensitive) or *Msp*I (methylation insensitive) (ER0511 and RR0541, Thermo Fisher Scientific). Blotting was done to a Hybond-XI membrane (GERPN203S, GE Healthcare) for 6 hours, and ultraviolet–cross-linked to the membrane with Strategene 1200 using auto cross-link setting (1200 μJ). Hybridization was done overnight at 65°C. The filter was exposed and analyzed on a Molecular Devices Storm 840 Scanner and Image Processor. Primer sequences for the generation of the ^32^P-labeled probes can be found in table S1.

### Flow cytometry

Single-cell suspensions of embryonic spleens were prepared by squeezing the tissues through a 35-μm nylon cell strainer (352235, Corning). Erythrocytes were stained with eFluor 450–conjugated TER-119 (48-5921-82, Thermo Fisher Scientific) in fluorescence-activated cell sorting (FACS) buffer [phosphate-buffered saline (PBS) with 2% bovine serum albumin (BSA) and 0.1% sodium azide] for 30 min at 4°C. Flow cytometry was performed using a FACSCanto II (BD Biosciences) and analyzed using FlowJo software v10.6.1 (Tree Star). The gating strategy is shown in fig. S2.

### Chromatin immunoprecipitation followed by sequencing

For chromatin preparation, the frozen spleen was smashed using a mortar and pestle (done in liquid nitrogen) and cross-linked with 1% formaldehyde (344198, Calbiochem) in 9 ml of 1% PBS for 15 min at room temperature (RT). Glycine (125 mM) was used to quench cross-linking for 5 min, and tissue was washed twice with cold PBS containing proteinase inhibitors (cOmplete, EDTA-free protease inhibitor cocktail, 5056489001, Roche). For lysis, 1 ml of cytoplasmic lysis buffer [10 mM tris (pH 8), 10 mM NaCl, 0.2% IGEPAL, and freshly added protease inhibitor cocktail (05056489001, Roche)] was used. Tissue was homogenized two times for 10 s (IKA model - T10 basic, at max speed – 6). Samples were spun down at 4000 rpm for 8 to 10 min at 4°C, and the pellets were resuspended in 1 ml of SDS lysis buffer [1% SDS, 50 mM tris-HCl (pH 8.1), 10 mM EDTA, and freshly added protease inhibitor cocktail]. Lysed samples were sheared by sonication (Diagenode Biorupter Pico, −30-s ON/30-s OFF, 15 to 25 cycles). Sheared chromatin was centrifuged at 12,000*g* for 15 min at 4°C to discard the pellets. Before use, the chromatin was diluted 10 times with NP buffer [150 mM NaCl, 50 mM tris-HCl (pH7.5), 5 mM EDTA, 0.5% NP-40, 1% Triton X-100, and freshly added protease inhibitor cocktail] to make the final SDS concentration lower than 0.1%. For histone ChIP, precleared chromatin was first incubated with 5 μg of antibodies H3K9me3 (39161, Active motif) or H3K27me3 (17-622, Millipore) at 4°C overnight. Protein A Sepharose beads (GE17528001, Sigma-Aldrich) blocked with BSA (1 mg/ml; 10848, Affymetrix) were added to pull down the antibody-chromatin complex. After immunoprecipitation, beads were washed with low-salt washing buffer [0.1% SDS, 1% Triton X-100, 2 mM EDTA, 20 mM tris-HCl (pH 8.1), and 150 mM NaCl], high-salt washing buffer [0.1% SDS, 1% Triton X-100, 2 mM EDTA, 20 mM tris-HCl (pH 8.1), and 500 mM NaCl], LiCl washing buffer [0.25 M LiCl, 1% NP40, 1% deoxycholate, 1 mM EDTA, and 10 mM tris-HCl (pH 8.1)], and two times with TE buffer [10 mM tris-HCl (pH 8.0) and 1 mM EDTA). Input DNA samples were extracted with phenol-chloroform-isoamyl alcohol (15593049, Thermo Fisher Scientific). For ChIP-seq, immunoprecipitated DNA was purified using phenol-chloroform-isoamyl alcohol, and DNA concentration was measured using Qubit. For library preparation, the Kapa Hyper Prep Kit (KK8504, 07962363001, Roche) was used, following the manufacturer’s instructions. Samples were sequenced at GenomeScan on a NovaSeq6000 with 150–base pair (bp) PE reads.

### Nuclei isolation (for snRNA-seq or ULI-ChIP-seq)

Nuclei were isolated from the embryonic cerebrum as previously described (*88*). Briefly, tissue was chopped, flash-frozen, and stored at −80°C. The Dounce homogenizer (2 ml), pestles, and all buffers [NIM1, 250 mM sucrose, 25 mM KCl, 5 mM MgCl_2_, and 10 mM tris (pH 8); NIM2 – NIM1, 1 μM dithiothreitol and protease inhibitor; homogenization buffer – NIM2, RiboLock (0.4 U/μl) (EO0384, Thermo Fisher Scientific)] were precooled. Tissue was homogenized in 1 ml of homogenization buffer with 5 strokes (loose pestle), followed by 15 strokes (tight pestle). Homogenate was filtered through a BD Falcon tube with a 35-μm nylon cell strainer cap (352235, Corning). Nuclei were counted by staining an aliquot with trypan blue and using TC10 Automated Cell Counter (Bio-Rad). Nuclei were resuspended in 1 ml of NIM2 buffer and the sample was centrifuged (500*g*, 5 min, 4°C). The washing step was repeated two to three times.

### ULI-ChIP-seq and ChIP-qPCR

ULI-ChIP-seq was performed as previously described (*89*). Briefly, around 1 million nuclei per IP were diluted in 50 μl of nuclear storage buffer [15% sucrose, 10 mM tris (pH 7.2), 70 mM KCl, 2 mM MgCl_2_, and proteinase inhibitors] and fragmented using MNase at 37°C for 7.5 min. Reaction was stopped with 6.6 μl of 100 mM EDTA and 6.6 μl of 1% Triton X-100/1% DOC and samples were diluted up to 200 μl with complete immunoprecipitation buffer [20 mM tris-HCl (pH 8.0), 2 mM EDTA, 15 mM NaCl, 0.1% Triton X-100, 1× EDTA-free protease inhibitor cocktail, and 1 mM phenylmethylsulfonyl fluoride (PMSF)]. Chromatin was precleared with 20 μl of a 1:1 mixture of protein A and protein G Dynabeads (10006D and 10007D, Life Technologies). Two micrograms of anti-H3K4me3 (17-614, Millipore), H3K27me3 (17-622, Millipore), or H3K9me3 (ab8898, Abcam) were first bound to 20 μl of a 1:1 mixture of protein A and protein G Dynabeads, and then incubated overnight at 4°C with precleared chromatin. The IP samples/beads were washed the next day twice with 200 μl of a low-salt wash buffer [20 mM tris-HCl (pH 8.0), 2 mM EDTA, 150 mM NaCl, 0.1% SDS, and 1% Triton X-100] and twice with a high-salt wash buffer containing [20 mM tris-HCl (pH 8.0), 2 mM EDTA, 500 mM NaCl, 0.1% SDS, and 1% Triton X-100]. DNA was eluted in 30 μl of a ChIP elution buffer (100 mM NaHCO3 and 1% SDS) for 1.5 hours at 65°C. The eluted DNA was purified by phenol-chloroform, precipitated with EtOH, and resuspended in 20 μl of Milli-Q. For library preparation, the Kapa Hyper Prep Kit (KK8504, 07962363001, Roche) was used, following the manufacturer’s instructions. Samples were sequenced at Macrogen on a NovaSeq6000 with 150-bp pair-end (PE) reads. For ChIP-qPCR, isolated DNA was directly used (in triplicate) on a C1000TM thermal cycler (Bio-Rad) with SYBR Green (170-8887, Bio-Rad). Primer sequences are provided in table S1.

### CTCF ChIP-qPCR

ChIP was performed as previously described (*74*), with minor modifications. Cerebrums from two animals per genotype were pooled and crosslinked in 1% formaldehyde/PBS [conatining 1× EDTA-free protease inhibitor cocktail (05056489001, Roche) and 1 mM PMSF, (93482, Sigma-Aldrich)] for 10 min and subsequently quenched with 125 mM glycine for 5 min at RT. After washing crossed-linked tissues twice with ice-cold PBS (containing 1× EDTA-free protease inhibitor cocktail), nuclei were isolated using the above-described procedure. Around 10^6^ nuclei were digested in 50 μl of lysis buffer [20 mM tris-HCl (pH 7.5), 70 mM NaCl, 1% Triton-X-100, 0.1% sodium deoxycholate, 0.1% SDS, and 1× EDTA-free protease inhibitor cocktail] using MNase at 37°C for 8 min [master mix as described in (*89*)]. For each IP, 5 × 10^6^ nuclei were used. Isolated DNA was directly used (in triplicate) on a C1000TM thermal cycler (Bio-Rad) with SYBR Green (170-8887, Bio-Rad). Primer sequences are provided in table S1.

### Whole-genome bisulfite sequencing

gDNA was isolated as described above and WGBS was performed at BGI (samples were sequenced on HiSeq X Ten). Raw sequences were filtered by quality with TrimGalore using default parameters. Reads with lengths smaller than 20 bps and error rates higher than 0.1 were discarded. Sequences were aligned to mm10 (transgene sequence was included in mm10 FASTA file) using the Bismark aligner v0.18 (*90*). To increase sensitivity, we used parameter “-N 1”, as well as –gzip –bowtie2. Duplicates were removed with Deduplicate Bismark (Bismark package). Methylation calling was performed by Bismark Methylation Extractor, using default parameters with the following exceptions: “–paired-end,” “–ignore_r2 2,” and “–bedgraph.” Correlation was calculated and plots were made using the Methylkit R package (*91*). Differentially methylated CpGs were calculated using DSS R package v2.3 (Bioconductor package) with the following parameters *P* < 0.05 and alpha > 0.15, and, subsequently, the annotation was done with ChIPseeker (*92*). For violin plots, the genome was divided in 1-kb windows [bedtools (*93*) was used to create 1-kb bins] and average methylation for each bin per genotype was calculated, then the bins were annotated using ChIPseeker, after which ggplot was used to create violin plots. Mouse (mm10) soloCpG (WCGW), HMD, and PMD annotations were obtained from previous publications (*17*) and bedtools was used for the intersection of the datasets. To visualize the distribution of methylation levels for soloCpGs located in HMDs and PMDs, Python libraries seaborn (v0.12.2) and matplotlib (v3.6.3) were used.

### ONT long-read sequencing adaptive sampling

gDNA was isolated as described above. DNA concentration was determined by Qubit using the Qubit High Sensitivity Kit, and size was determined on a Femto Pulse System (Agilent Technologies). ONT sequencing libraries were prepared using the gDNA ligation kit (SQK-LSK114) and sequenced using the vendor’s instructions. Briefly, 600 ng of gDNA was used as input and yielded approximately 20 fmol of the library to load on a PromethION Flowcell (FLO-PRO114M) and sequenced on a PromethION 24 device (A100). Adaptive sampling in MinKNOW (v22.10.7) was enabled during the sequencing of these samples using fast basecalling and a BED file was provided for the regions of interest while mm10 of the mouse genome was used as a reference for the enrichment. After 20 hours, the run yielded around 400k reads which accounts for 3.53 Gb with an N50 of 28 kb. The basecalling with modification was done after the adaptive sampling run was completed using Guppy version 6.3.9 with model dna_r10.4.1_e8.2_400bps_modbases_5mc_cg_sup_prom.cfg. The BAM file containing the fastq reads and the modifications were mapped against mm10 using SAMtools (version 1.13) with operation fastq and flags -TMM,ML and piped to Minimap2 (version 2.24) where no secondary mapping was allowed. The output results in a sam file which also contains the modification information. The sam files were converted to BAM files, sorted and indexed using SAMtools. Methylartist (*88*) was used to visualize methylation patterns across various genomic regions, using the commands ‘locus’ and ‘region’. Default parameters for smooth window size were used and are indicated in the figure panels.

### ChIP-seq analyses

After preprocessing with TrimGalore to remove low-quality reads, reads were aligned to the mouse genome (mm10) using bowtie2 v2.3.4.2 (*94*) with parameters -very sensitive. Peaks were called using MACS2 (*95*) for H3K4me3 ChIP-seq (-f BAMPE, -g mm) or SICER (*96*) for H3K9me3 (parameters: W200 and G600) and H3K27me3 (parameters: W1000 and G4000). For ChIP-seq, peaks were called using input fractions as control. For differential peak calling, DiffBind was used (for H3K4me3, default parameters; for H3K9me3 and H3K27me3, summit FALSE). Peaks were annotated using ChIPseeker (*92*) (version 1.22.1). To generate tracks, heatmaps, and profile plots, deepTools (*97*) version 3.1.3 was used. For the heatmaps and profile plots a bin size of 100 bps was chosen.

### Genome-wide analysis of DNA methylation and histone marks at repetitive elements

To generate profile plots over repetitive elements, deepTools (*97*) and the UCSC RepeatMasker track (mm10) were used. For the heatmaps, coverage and BAM files generated during the analyses of the WGBS and H3K9me3 ChIP-seq data were used. X and Y chromosomes were filtered using SAMtools (v1.10) and all samples were down-sampled to maintain 25 M reads per sample using the function *DownsampleSam* from Picard (v2.18.7). For heatmaps, methylation percentage at individual repeats was measured by overlapping the methylation value with repeat masker coordinates using the function *bedtools intersect* (v2.30) (*93*). H3K9me3 coverage was measured over individual TEs extracted from the UCSC repeat masker using the function *bedtools multicov* (v2.30). Heatmaps showing the average percentage of methylation and H3K9me3 enrichment over input at TEs of interest were generated in R using *pheatmap* (v1.0.12) and *dplyr* (v0.8.3) packages.

### Bulk RNA-seq analysis

Total RNA was isolated as described above and RNA-seq (rRNA depleted) was performed at BGI. Libraries were sequenced with 150-bp PE reads on a NovaSeq6000. Adapters were removed by TrimGalore v0.4.5 (www.bioinformatics.babraham.ac.uk/projects/trim_galore/) using default parameters for paired-end Illumina reads and reads <20 bps and/or error rate (TrimGalore option “-e”) higher than 0.1 were discarded. The remaining reads were mapped to the mm10 reference genome using STAR aligner v2.5.1 (*98*) using default parameters with the following exceptions: “–outputMultimapperOrder random” and “–twopassMode basic.” Before quantification, duplicated reads were marked with Picard tools v2.17 (*99*). Quantification was done by HTSeq-count v0.91 (*100*), using the GENCODE MV16 annotation with the option “–stranded no.” Statistical analysis was done using DESeq2 v1.2.0 (*101*) (R package). For TE analysis, trimmed reads were mapped to the mm10 reference genome using STAR aligner v2.7.0e with the same parameters as above for unique mapping. The annotations for TEs were obtained from http://labshare.cshl.edu/shares/mhammelllab/www-data/TEtranscripts/TE_GTF/GRCm38_Ensembl_rmsk_TE.gtf.gz.

### Single-nucleus RNA-seq analysis

snRNA-seq libraries were obtained using Chromium Next GEM Single Cell 5′ Library & Gel Bead Kit v1.1 (10x Genomics, PN-1000165) chemistry and Chromium Next GEM Chip G Single Cell Kit (10x Genomics, PN-1000120). Two libraries were prepared per genotype. Samples were sequenced on an Illumina NovaSeq 6000 aiming for 2500 reads per nucleus. UMI (unique molecular identifiers) containing reads were mapped and counted with Cell Ranger 6.0.1 using a custom pre-mRNA GTF built on mm10 to include intronic reads. Because of a Cell Ranger annotation error, *Pcdha10* and *Pcdha11* read counts were combined (*Pcdha10_Pcdha11*) throughout the analysis. A mean of 205,514,987 reads (SD = 16,565,352) were sequenced for each snRNA library corresponding to a mean of 23,932 reads per nucleus. The mean sequencing saturation was 70% (SD = 5%). Datasets were processed individually using Seurat v4.1.0 to filter out low-quality nuclei (Features > 1000, Features < 7500, Percentage of Mitochondrial genes < 5%). Filtered libraries were normalized using « LogNormalize » and scale factor = 10000. Clustering was performed for all libraries using the Seurat functions FindNeighbors() and FindClusters() with the respective parameters dims = 50 and resolution = 0.5. UMAP representations were created using the Seurat function RunUMAP(). Read counts for individual *Pcdha* isoforms were extracted from the Seurat object from each file using the Seurat function GetAssayData(). Before labeling, cell cycle gene expression was regressed according to (*102*). Individual nuclei identity was assigned on the basis of neuronal marker gene expression (*31*) and added to the Seurat object metadata. To remove batch effects, samples were integrated using the functions FindIntegrationAnchors() and IntegrateData() (*103*). Graphs and heatmaps were made using ggplot (v3.3.5) and pheatmap (1.0.12).

To perform differential gene expression per cell type, BAM files were generated for each cell type by filtering the output of Cell Ranger, using the barcodes linked to the cell types identified, using Sinto (0.7.5) (*104*). Count tables were generated for each cell type using featureCounts6 (2.0.1). The differential expression was measured using DESeq2 (1.34.0) (*101*) with genes considered as differentially expressed with *P*adj ≤ 0.01 and −1.5 ≤ log2FC ≤ 1.5. Maplots were generated using the function ggmaplot() from ggpubr (0.4.0) (https://cloud.r-project.org/package=ggpubr).

### Statistical analyses

Statistical significance of body weight and quantitative data was determined using Student’s *t* test. *F* test was used to test whether variance was significantly different between WT and mutant groups. All statistical analyses were performed in GraphPad Prism.

### Sample size determination

No statistical methods were used to predetermine the sample size. We always used as many samples per genotype as possible. Experiments, where possible, were performed at least in two independent measurements. All sample sizes are indicated in the figures and/or figure legends.

### Public datasets

ChIP-seq data for H3K4me3 in the spleen (GSM769036) and Pol2r (GSM722993) were obtained from EncodeProject (*105*) and CistromeDB (*106*). Coordinates for cLADs were obtained from GEO (GSE17051) and were transformed to mm10 coordinates using the UCSC liftover tool. Coordinates of mouse common HMD, PMD, and solo-WCGW were downloaded from (*107*) GitHub (https://zwdzwd.github.io/pmd). Repli-chip in mesodermal cell (ENCFF001JUT) was obtained from EncodeProject. A list of curated ICRs was obtained from (*108*).
