# Supplementary material for: CDCA7-associated global aberrant DNA hypomethylation translates to localized, tissue-specific transcriptional responses
Source: Sci Adv. 2024 Feb 9;10(6):eadk3384. doi: 10.1126/sciadv.adk3384 (PMC10857554; doi:10.1126/sciadv.adk3384)
Supplement: Supplementary file 1 — Figs. S1 to S20 Table S1 Legends for data S1 and S2 [file sciadv.adk3384_sm.pdf]

Science Advances
AAAS

Supplementary Materials for

CDCA7-associated global aberrant DNA hypomethylation translates to localized, tissue-specific transcriptional responses

Maja Vukic *et al.*

Corresponding author: Lucia Daxinger, l.clemens-daxinger@lumc.nl

Sci. Adv. **10**, eadk3384 (2024)
DOI: 10.1126/sciadv.adk3384

The PDF file includes:

Figs. S1 to S20
Table S1
Legends for data S1 and S2

Other Supplementary Material for this manuscript includes the following:

Data S1 and S2

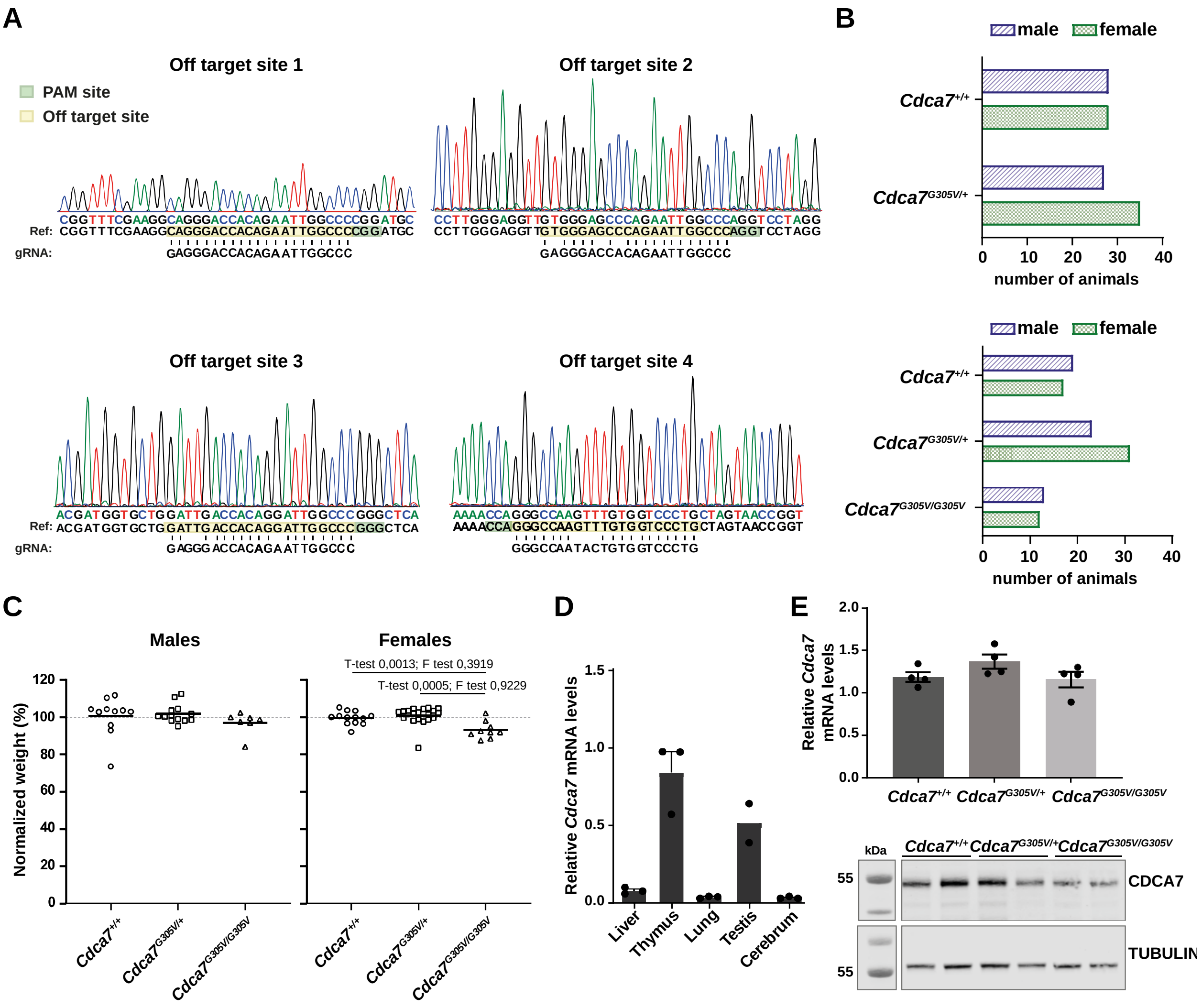

Fig. S1. CDCA7 mouse model.

(A) Representative Sanger sequencing traces from *Cdca7*$^{G305V/+}$ mice showing no mutations at the 4 top predicted CRISPR off target sites, the reference sequence is displayed for comparison. (Genomic location and primers used can be found in Table S1).

(B) (Top) Bar chart showing number of male and female offspring observed at weaning (P21) after WT and heterozygous intercross. Data collected from 12 litters. (Bottom) Bar chart showing number of male and female offspring at weaning (P21) after heterozygous intercross (animals correspond to Figure 1B divided by sex).

(C) Scatter plots showing normalized weights of P21 male (right) and female (left) animals. Weights for each litter were normalized to the average weight of WT in that litter. Each data point represents an individual. 8 litters were used for the analysis; T-test and F-test were performed.

(D) RT-qPCR analysis of relative *Cdca7* mRNA levels in different tissues at P21 (data are normalized to *β-actin*; n = 2 or 3 biological replicates; data are presented as mean with black dots as individual values; Error bar - SEM).

(E) (top) RT-qPCR analysis of relative *Cdca7* mRNA levels in P21 thymus (Data are normalized to *β-actin*; n = 4 biological replicates; Data are presented as mean with black dots as individual

values; Error bar - SEM). (bottom) Western blot showing CDCA7 protein in P21 thymus; two biological replicates per genotype; TUBULIN was used as a loading control.

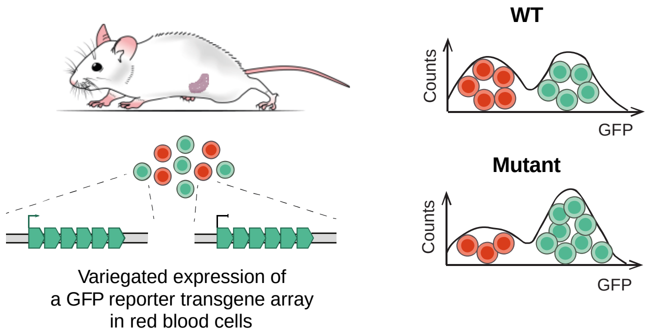

embryo	% GFP+ erythrocytes	MFI
Cdca7$^{+/+}$	46 ± 4	1087 ± 246
Cdca7$^{G305V/G305V}$	63 ± 3	2480 ± 457

3 weeks	% GFP+ erythrocytes	Ref
Dnmt1$^{+/+}$	53 ± 3	(10)
Dnmt1$^{MD2/+}$	76 ± 3	(10)
Dnmt1$^{MD32/+}$	80 ± 3	(12)
Uhrf1$^{MD40/+}$	75 ± 1	(12)
Dnmt3b$^{MD14/+}$	77 ± 4	(13)
Dnmt3b$^{MD14/MD14}$	92 ± 1	(13)

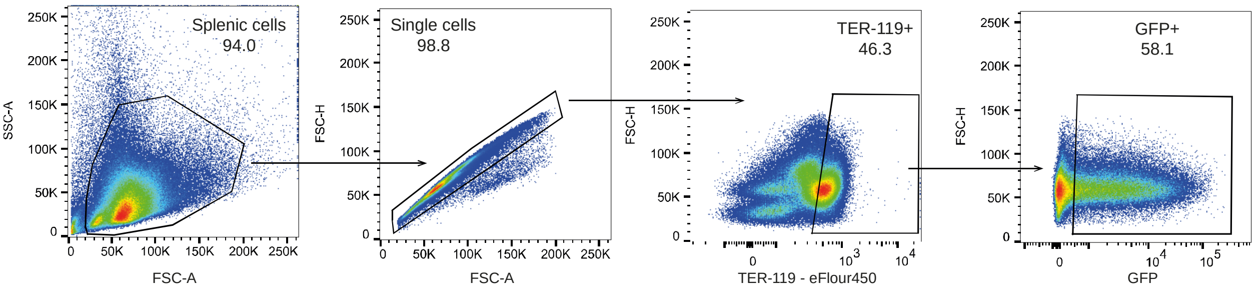

Fig. S2. The *Cdca7*^{G305V} mutation increases the percentage of erythrocytes expressing GFP. (Top left) The GFP transgene array is expressed in erythrocytes in a variegated manner. WT and mutants are classified based on the percentage of red blood cells expressing GFP. (Top right) Tables showing average percentage of GFP positive (GFP+) erythrocytes and mean fluorescent intensity (MFI) in WT (n=7) and *Cdca7*^{G305V} homozygous embryonic spleen (n=6), as well as previously reported *Dnmt1*, *Uhrf1* and *Dnmt3b MommeD* mutants at three weeks. (Bottom) Representative flow cytometric plots for the gating of GFP positive erythrocytes in embryonic spleen.

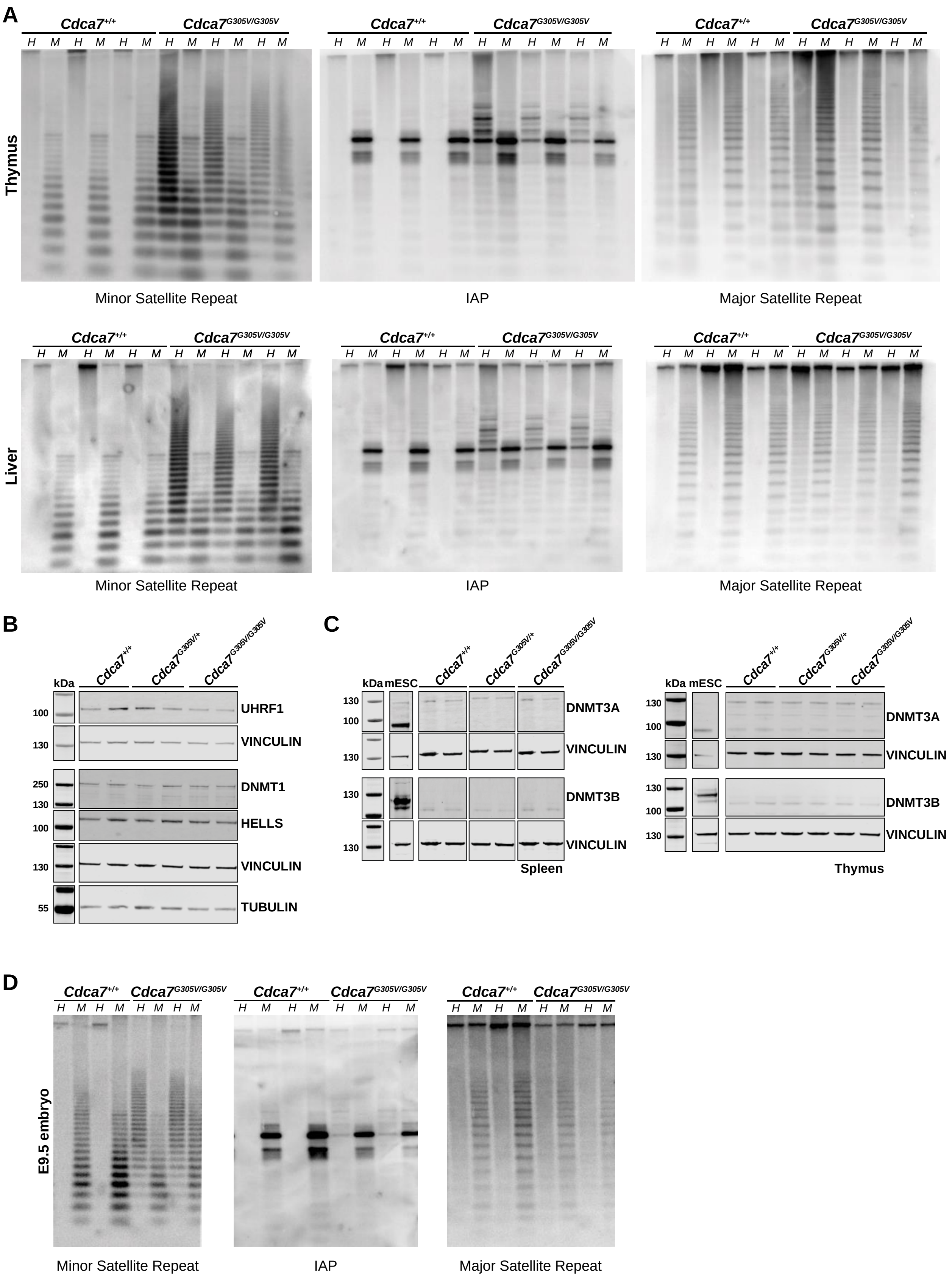
A
Cdca7+/+
Cdca7G305V/G305V
H M H M H M H M H M H M
Thymus
Minor Satellite Repeat
IAP
Major Satellite Repeat
Liver
B
kDa
Cdca7+/+
Cdca7G305V/+
Cdca7G305V/G305V
100
130
250
55
UHRF1
VINCULIN
DNMT1
HELLS
TUBULIN
C
mESC
DNMT3A
DNMT3B
Spleen
Thymus
D
E9.5 embryo

Fig. S3. CDCA7 is required to maintain repeat DNA methylation levels in adult tissues and embryos.

(A) Southern blots showing minor satellite repeat, IAP and major satellite repeat methylation levels in P21 (top) thymus and (bottom) liver. Genomic DNA was digested with *Hpa*II (*H*; methylation sensitive) or *Msp*I (*M*; methylation insensitive). Three biological replicates per genotype.

(B) Western blot showing DNMT1, UHRF1 and HELLS protein levels in P21 thymus, two biological replicates per genotype. TUBULIN and/or VINCULIN were used as loading controls.

(C) Western blot showing DNMT3A and DNMT3B protein levels in P21 spleen and thymus, two biological replicates per genotype. TUBULIN and/or VINCULIN were used as loading controls and mESCs as a positive control for DNMT3A/B expression.

(D) Southern blots showing minor satellite repeat, IAP and major satellite repeat methylation levels in E9.5 embryos. Genomic DNA was digested with *Hpa*II (*H*; methylation sensitive) or *Msp*I (*M*; methylation insensitive). Two biological replicates per genotype.

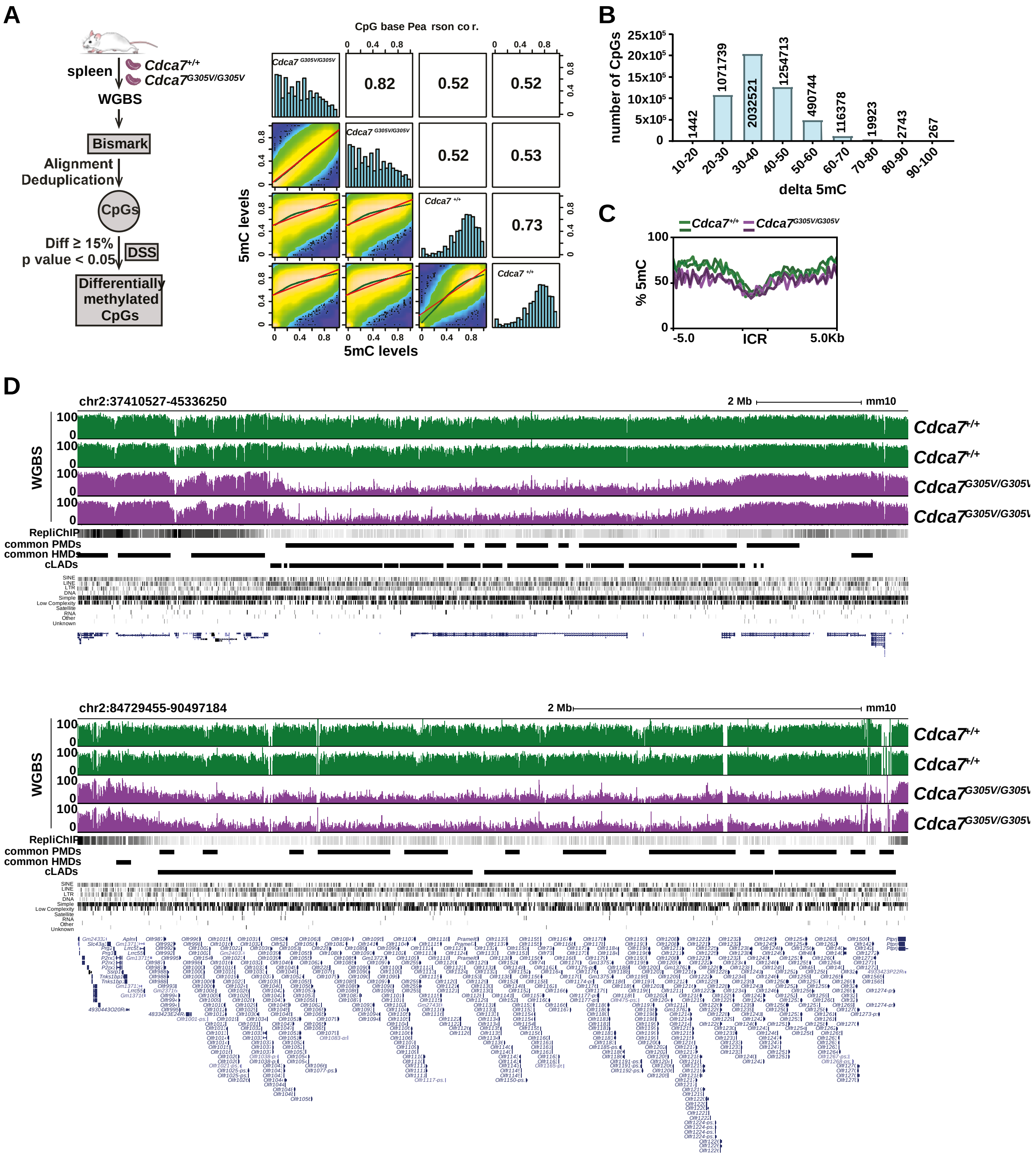

Fig. S4. CDCA7 is required to maintain global DNA methylation levels in spleen.

(A) (left) Schematic representation of the pipelines used for WGBS analyses. (right) Density scatter plots showing correlation of CpG methylation scores between four WGBS samples. The density of points increases from blue to yellow. Numbers in the upper right corner indicate Pearson's correlation coefficient between samples. Bar plots in diagonal squares show proportion of CpGs having 0 to 100 % methylation in each sample. The X-axis was divided into 20 individual 5 % interval bins. WT samples show a higher proportion of methylated CpGs,

whereas *Cdca7*^{G305V} homozygous samples skew left, indicating an increased percentage of lowly methylated CpGs.
(B) Bar chart showing the number of CpGs with Δ % 5mC (WT – *Cdca7*^{G305V} homozygous) indicated on the X-axis.
(C) Profile plots showing CpG methylation levels over 17 ICR (https://github.com/zhou-lab/ImprintingAnno), and flanking 5 kb regions (methylation levels were calculated over 200 bp bins).
(D) Genome browser view depicting WGBS tracks over two regions on chromosome 2. (top) Representative gene poor hypomethylated region (zoom in from Figure 3D), and (bottom) representative hypomethylated gene cluster region (zoom in from Figure 3D) – in this example one of the *olfactory receptor* clusters. Two replicates per genotype are shown. RepeatMasker and RefSeq annotations are shown below the tracks; Replication timing in mesodermal cell (ENCFF001JUT) is shown below the methylation. Black bars – common PMDs, common HMDs (17) and cLADs (GSE17051).

A

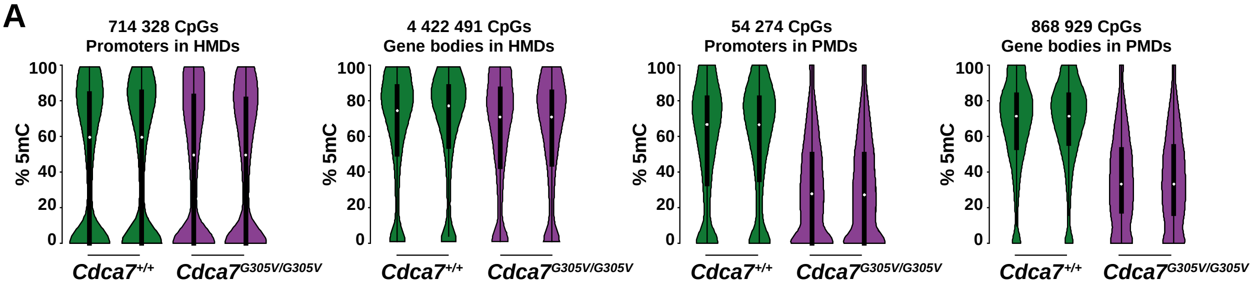

B

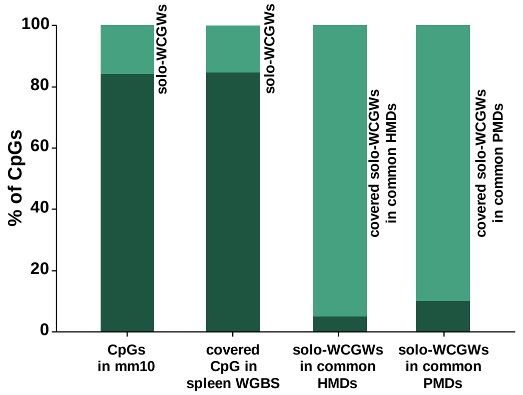

CpGs in mm10	~ 21.000.000
CpGs covered in all 4 WGBS data sets	19.185.097
solo-WCGW in mm10	3.308.350
solo-WCGW in common HMDs mm10	694.829
covered solo-WCGW in common HMDs	662.080
solo-WCGW in common PMDs mm10	743.327
covered solo-WCGW in common PMDs	668.812

Fig. S5. CDCA7 preferably promotes methylation of genes located in PMDs.

(A) Violin plots showing CpG methylation at promoters and gene bodies located in common (left) HMDs or (right) PMDs. Two biological replicates per genotype are shown. Number indicates number of CpGs plotted.

(B) (Left) Bar charts showing percentage of solo-WCGW sites present in mm10 genome assembly, covered in all WGBS spleen data sets, present in common HMDs or PMDs. (Right) Table showing the numbers used to generate bar charts on the left.

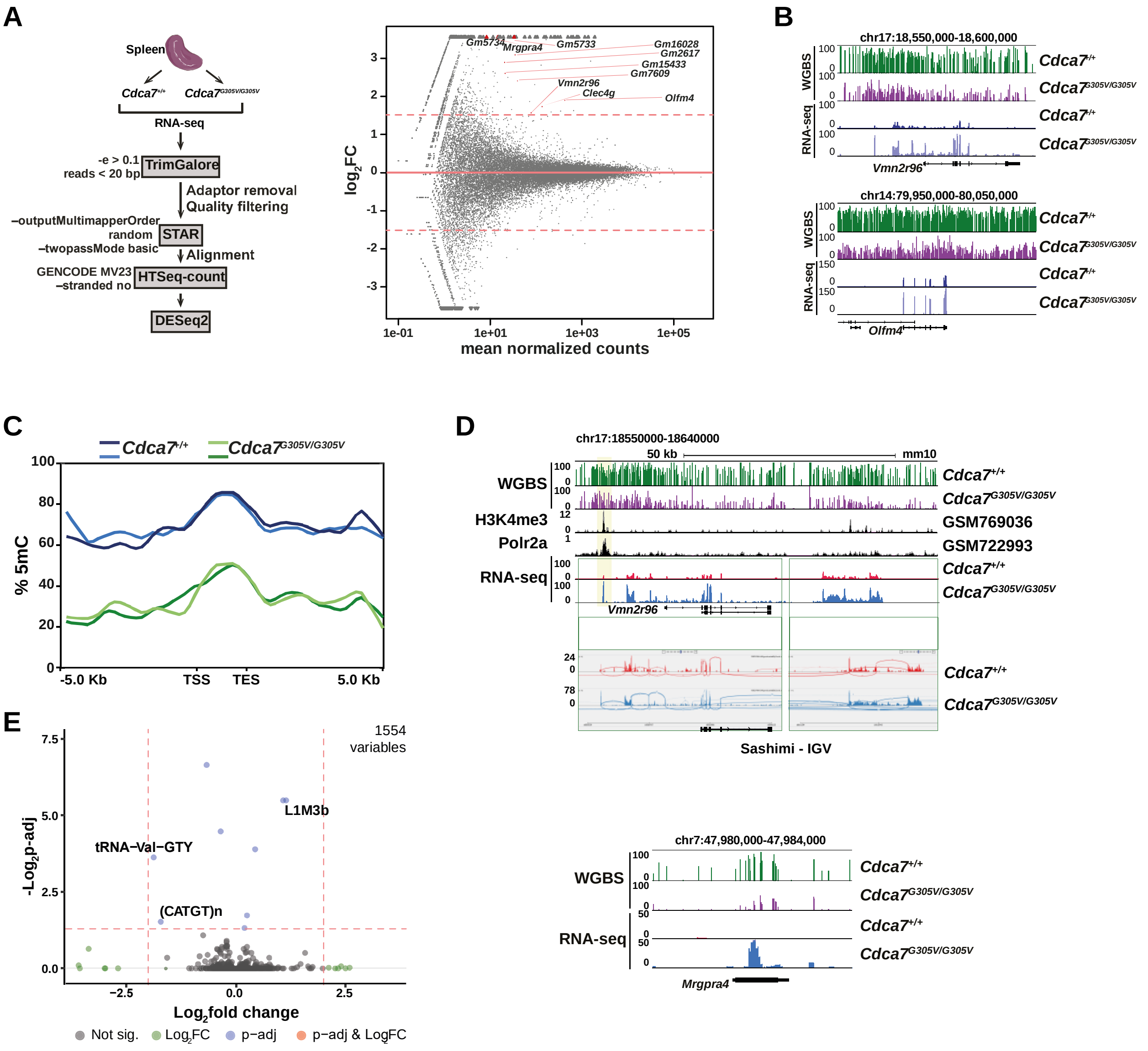

Fig. S6. Aberrant hypomethylation is associated with modest upregulation of lowly-expressed genes in spleen of *Cdca7*^{G305V} homozygotes.

(A) (Left) Schematic representation of the RNA-seq analysis pipeline. (Right) MA plot of normalized RNA-Seq data. Red dots plus names indicate differentially expressed genes (p-adj < 0.05 and $\log_2$FC > 1.5) between *Cdca7*$^{G305V/G305V}$ and WT spleen. The *y*-axis shows $\log_2$FC and the *x*-axis the average log intensity (mean expression).

(B) Genome browser screenshot of WGBS and RNA-seq tracks depicting two loci that are hypomethylated and differentially expressed (*Vmn2r96* – top; *Olfm4* - bottom) in *Cdca7*^{G305V} homozygotes. (Representative WT and *Cdca7*^{G305V} homozygous samples are shown.)

(C) Profile plot showing DNA methylation levels in *Cdca7*$^{G305V/G305V}$ compared to WT spleen over gene bodies of the 8 differentially expressed genes and 5 kb flanking regions.

(D) Genome browser screenshots of examples of hypomethylated and differentially expressed genes and their complex genomic structures. (Top) *Vmn2r96* locus: H3K4me3, Polr2a, and RNA-seq datasets suggest that the transcription start sites and transcripts in the spleen differ

from the current mm10 annotation. Sashimi plot visualization of aligned RNA-seq reads in IGV shows splice junctions of the transcripts. (Bottom) *Mrgpra4* locus: WGBS and RNA-seq reads in WT and *Cdca7*^{G305V} homozygous spleen are shown. (Representative WT and *Cdca7*^{G305V} homozygous samples are shown.)

(E) Volcano plot showing log_2FC in transposable element (TE) expression between WT and *Cdca7*^{G305V} homozygotes. TE expression levels of three biological replicates were measured and $log_2FC > 2$ and p-adj < 0.05 were considered significant. Reads were allowed to multimap. The *y*-axis shows $-log_2$p-adj and the *x*-axis log_2FC.

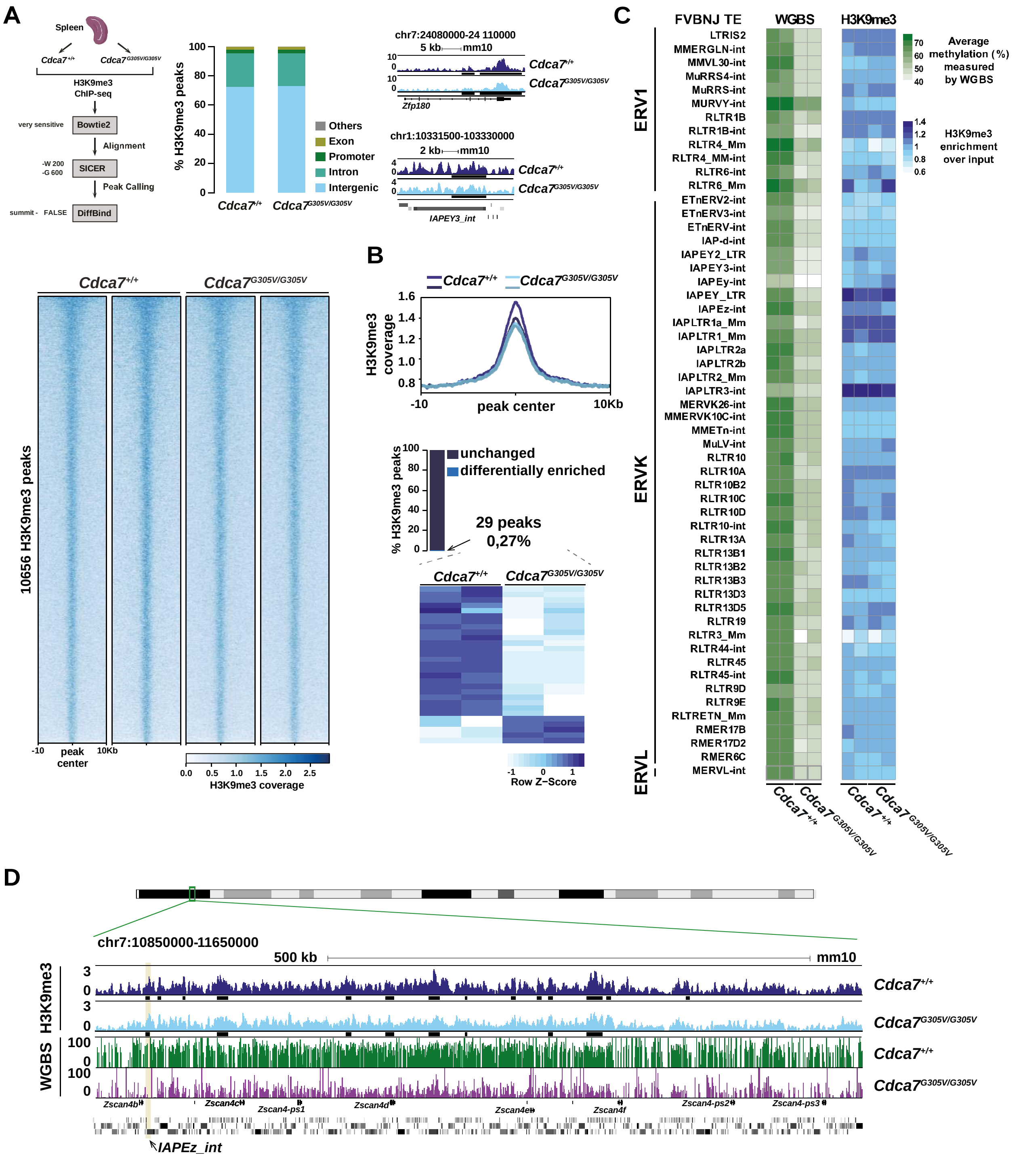

Fig. S7. CDCA7 is required for DNA methylation at H3K9me3 marked genomic sites but dispensable for H3K9me3 deposition.

(A) (Top left) Schematic representation of the H3K9me3 ChIP-seq analysis pipeline. (middle) Stacked bar chart showing proportion of H3K9m3 peaks with respect to their genomic annotation. (right) Genome browser screenshots depicting H3K9me3 levels over representative loci enriched for H3K9me3: *zinc finger protein 180* (*Zfp180*) gene and IAP elements. (Representative WT and *Cdca7*^{G305V} homozygous samples are shown. Black rectangles below

H3K9me3 tracks – H3K9me3 peaks.) (Bottom) Heatmaps showing H3K9me3 coverage over H3K9me3 peak center and 10 kb flanking regions (H3K9me3 levels were calculated over 10 bp bins).

(B) (top) Profile plot showing H3K9me3 from WT and *Cdca7*^{G305V} homozygous P21 spleen over center of the 10656 consensus H3K9me3 peaks and flanking 10 kb regions. (middle) Stacked bar chart indicating proportions of differentially enriched and unchanged H3K9me3 peaks (calculated by DiffBind, default settings - DEseq2, FDR $\leq$ 0.05). (bottom) Heatmap showing average H3K9me3 levels at the 29 differentially enriched loci in two WT and two *Cdca7*^{G305V} homozygotes.

(C) Heatmap of average CpG methylation and H3K9me3 levels over different endogenous retrovirus families.

(D) Genome browser view of H3K9me3 and WGBS methylation profiles over the *Zscan* gene cluster on chromosome 7. (Representative WT and *Cdca7*^{G305V} homozygous samples are shown. Black rectangles below H3K9me3 tracks – H3K9me3 peaks; RefSeq and Repeat masker annotations are shown below the tracks; Yellow shading - IAP covered by H3K9me3).

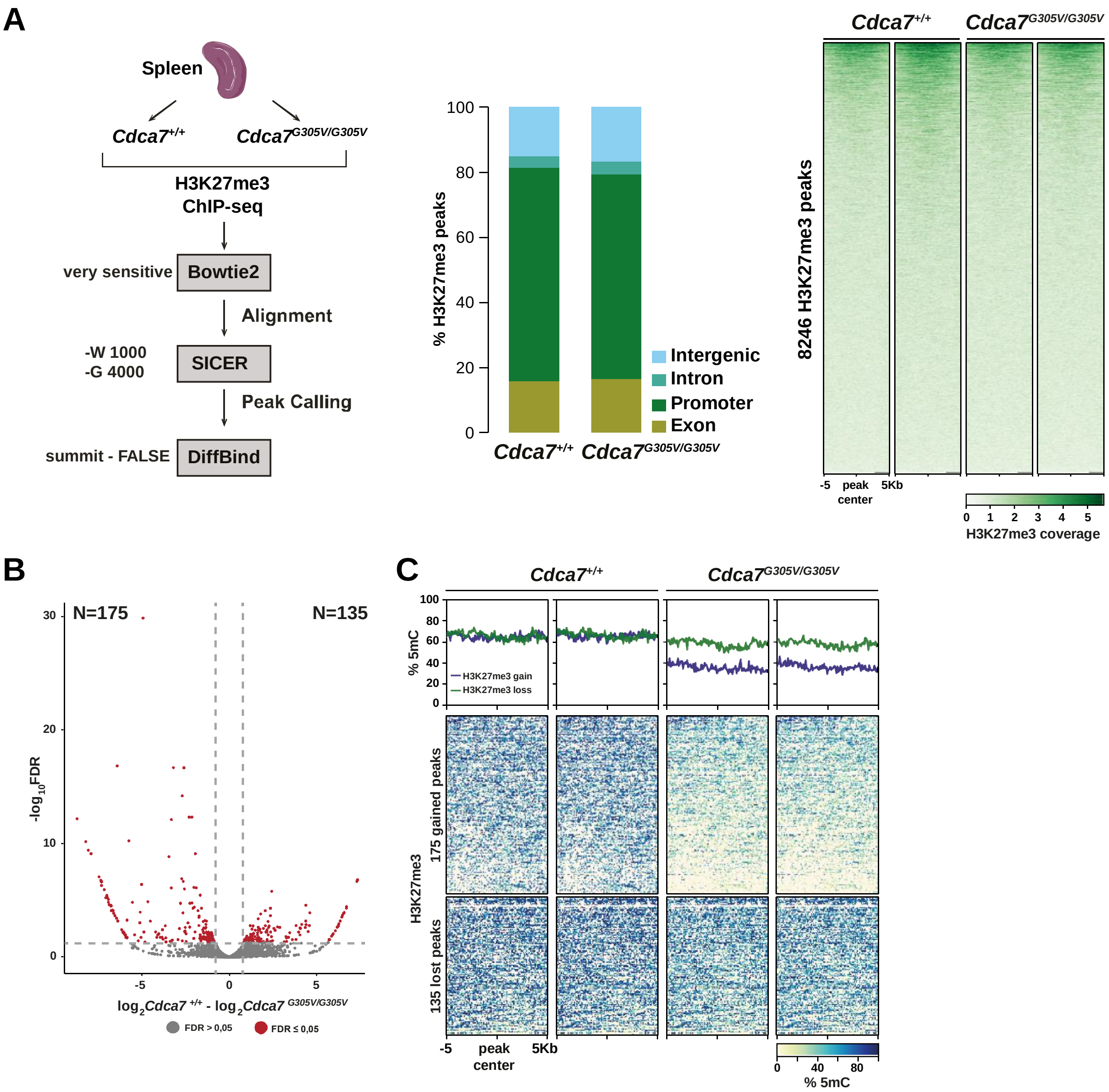

Fig. S8. Hypomethylation is accompanied by changes in H3K27me3 enrichment in *Cdca7*^{G305V} homozygous spleen.

(A) (left) Schematic representation of the H3K27me3 ChIP-seq analysis pipeline. (middle) Stacked bar chart showing proportion of H3K27me3 peaks with respect to their genomic annotation. (right) Heatmaps showing H3K27me3 coverage over H3K27me3 peak center and 5 kb flanking regions (H3K27me3 levels were calculated over 10 bp bins).

(B) Volcano plot showing H3K27me3 peaks that are differentially enriched (red dots) in *Cdca7*$^{G305V/G305V}$ spleens identified by DiffBind with default settings (DEseq2, FDR ≤ 0.05). The *y*-axis shows -log$_2$FDR and the *x*-axis log$_2$FC.

(C) DNA methylation levels over the center and 5 kb flanking regions of differentially enriched H3K27me3 peaks (gained or lost) depicted by profile plots and heatmaps (DNA methylation levels were calculated over 100 bp bins).

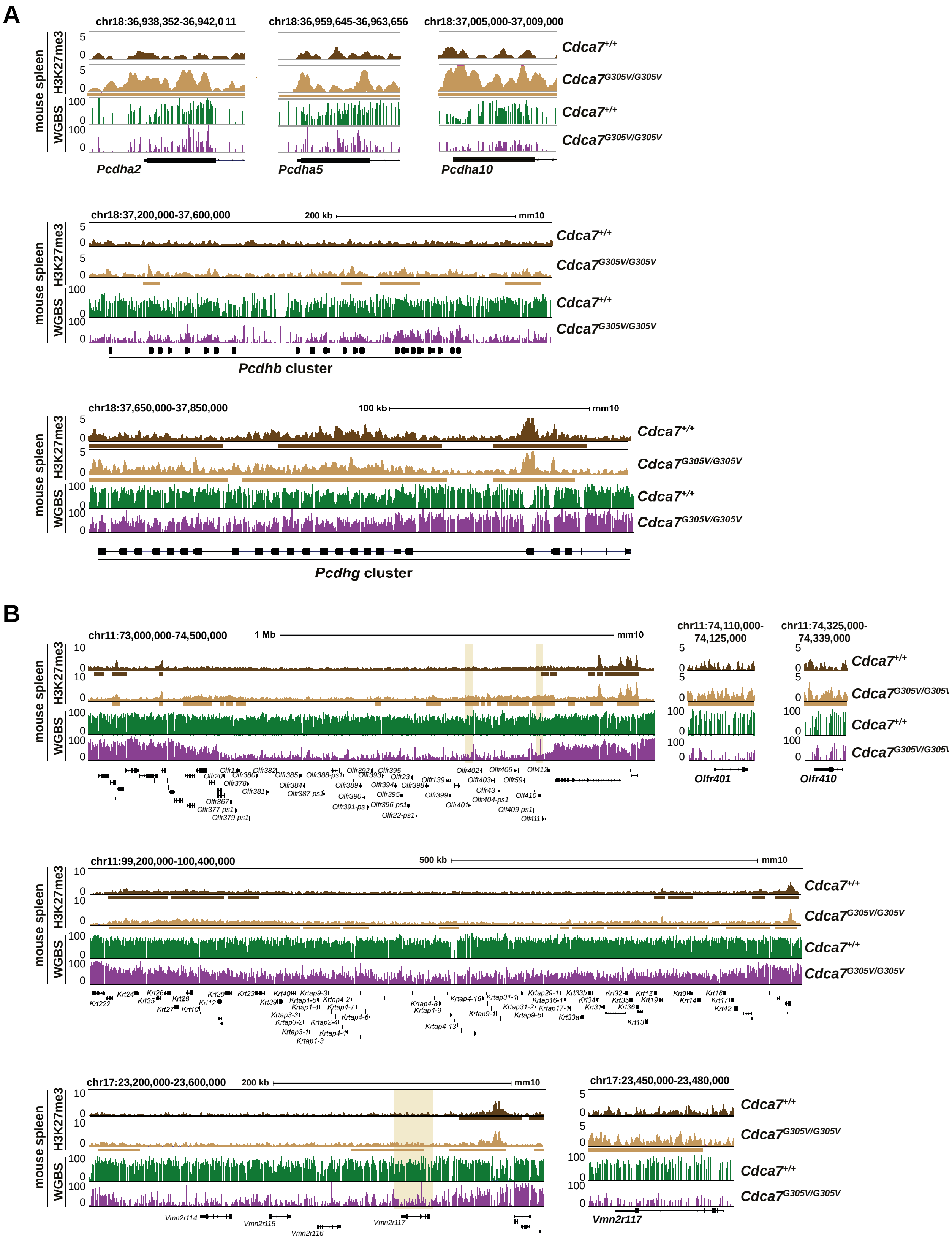

A
chr18:36,938,352-36,942,0 11
chr18:36,959,645-36,963,656
chr18:37,005,000-37,009,000
mouse spleen
H3K27me3
WGBS
Cdca7+/+
Cdca7G305V/G305V
Pcdha2
Pcdha5
Pcdha10
chr18:37,200,000-37,600,000
200 kb
mm10
Pcdhb cluster
chr18:37,650,000-37,850,000
100 kb
Pcdhg cluster
B
chr11:73,000,000-74,500,000
1 Mb
chr11:74,110,000-74,125,000
chr11:74,325,000-74,339,000
Olfr401
Olfr410
chr11:99,200,000-100,400,000
500 kb
chr17:23,200,000-23,600,000
chr17:23,450,000-23,480,000
Vmn2r114
Vmn2r115
Vmn2r116
Vmn2r117

Fig. S9. Hypomethylation is accompanied by increased H3K27me3 at gene clusters in *Cdca7*^{G305V} homozygous spleen.

(A) (top) Zoomed in genome browser view of H3K27me3 and DNA methylation levels over *Pcdha2*, *a5* and *a10* promoters. (middle) Genome browser screenshots of the 22 clustered *Pcdhb* and (bottom) 21 clustered *Pcdhg* genes. (Representative WT and *Cdca7*^{G305V} homozygous samples are shown. Rectangles below H3K27me3 tracks – H3K27me3 peaks; RefSeq annotation is shown below the tracks).

(B) Genome browser screenshots showing representative hypomethylated regions that gain H3K27me3 in *Cdca7*$^{G305V/G305V}$ spleen. Depicted are the (top) *Olfactory receptor* gene cluster on chromosome 11, (middle) the *Keratin* gene cluster on chromosome 11 and (bottom) the *Vomeronasal receptor* gene cluster on chromosome 17. (Representative WT and *Cdca7*^{G305V} homozygous samples are shown. Rectangles below H3K27me3 tracks – H3K27me3 peaks; RefSeq annotation is shown below the tracks. Yellow shaded regions are zoomed in and shown on the right).

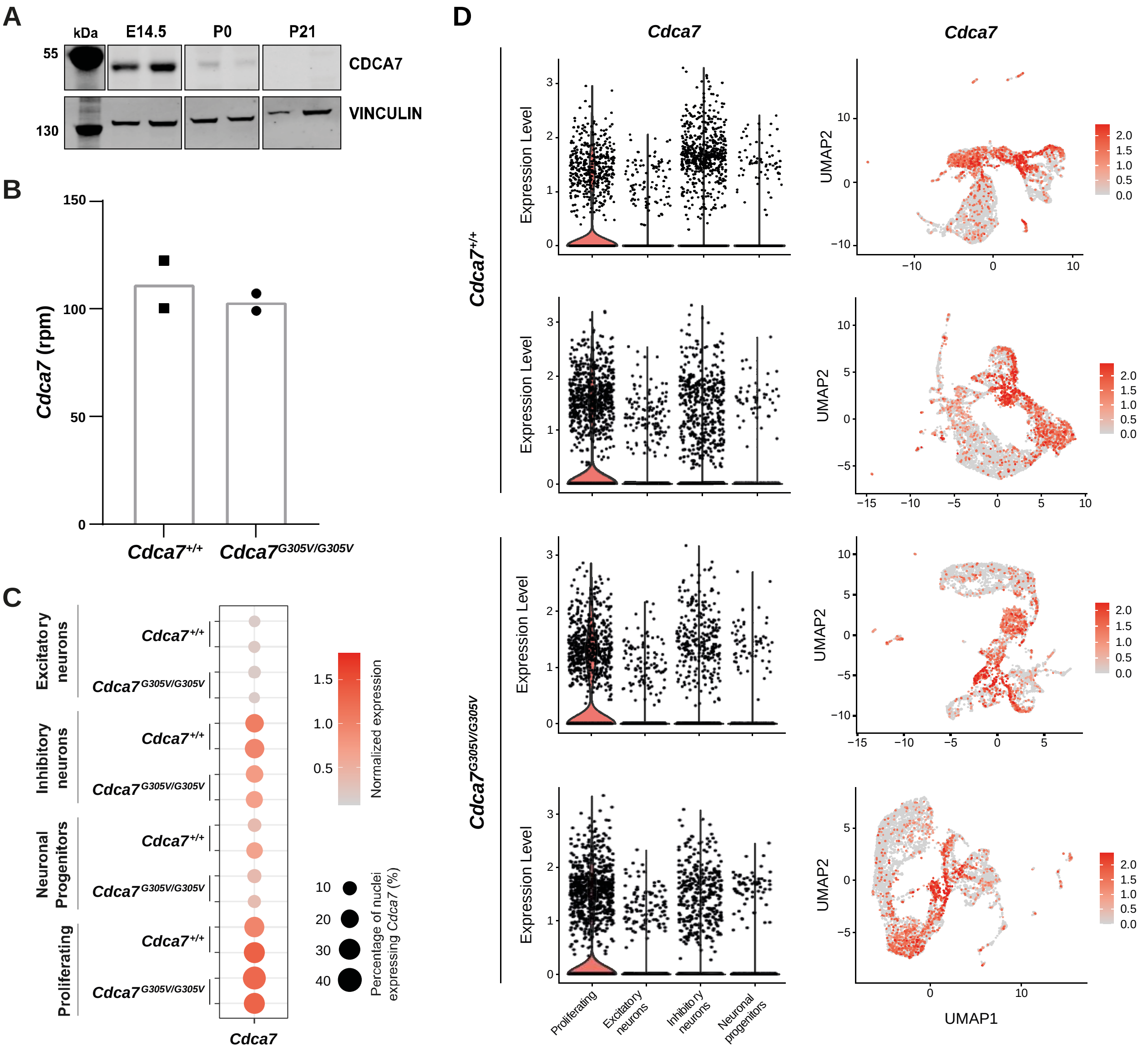

Fig. S10. CDCA7 protein and mRNA levels in E14.5 cerebrum.

(A) Western blot showing CDCA7 protein levels in E14.5, P0 and P21 WT cerebrum. Two biological replicates per developmental time point. VINCULIN was used as loading control.
(B) *Cdca7* mRNA levels determined by pseudo bulk analysis of snRNA-seq and represented in rpm (reads per million). Data are presented as mean with black dots as individual values.
(C) Dot plot showing normalized *Cdca7* mRNA expression levels in different cell types in WT and *Cdca7*^{G305V} homozygous E14.5 cerebrum (n=2 biological replicates/genotype).
(D) (Left) Violin and (right) feature plots of WT and *Cdca7*^{G305V} homozygous E14.5 cerebrum samples (n=2/genotype) showing nuclei expressing *Cdca7*.

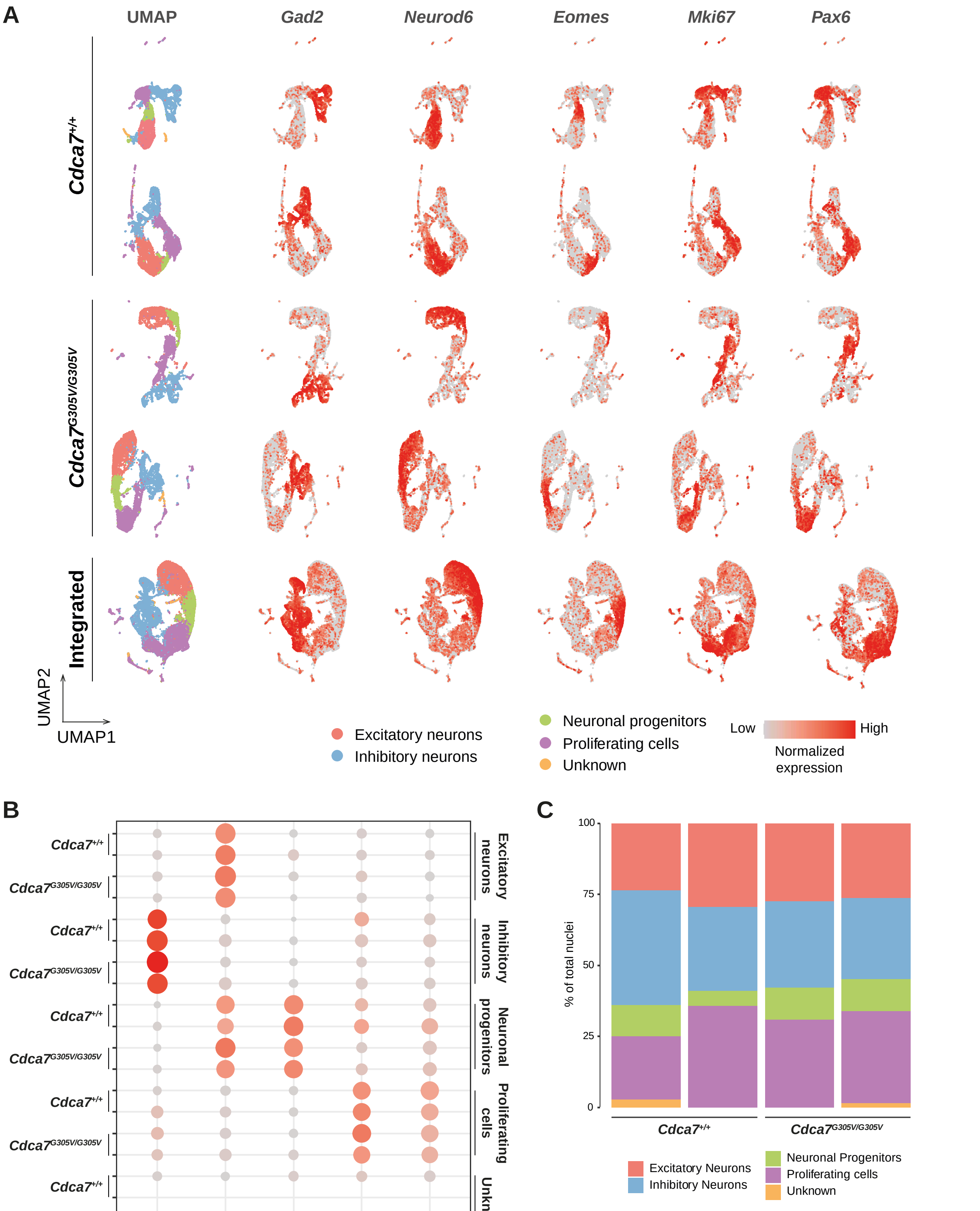

		Excitatory neurons	Inhibitory neurons	Proliferating cells	Neuronal progenitors	Unknown	Total nuclei
Cdca7+/+	rep1	1665	2855	1566	769	201	7056
	rep2	1971	1984	2393	344	0	6692
*Cdca7*G305V/G305V	rep1	1433	1576	1603	587	0	5199
	rep2	1838	1986	2256	792	104	6976

Fig. S11. snRNA-seq – annotation of different cell populations.

(A) (left panel) UMAP representation showing WT, *Cdca7*$^{G305V/G305V}$ and integrated E14.5 cerebrum samples (n=2 biological replicates/genotype) where single nuclei are colored by assigned cell type. (right panel) Feature plots showing single nuclei expressing the designated neuronal marker genes (in red) used for cell type annotation.

(B) Dot plot showing normalized marker gene expression levels used for cell type annotation in WT and *Cdca7*^{G305V} homozygous E14.5 cerebrum (n=2 biological replicates/genotype). Nuclei populations annotated as "unknown" do not express any of the neuronal marker genes.

(C) (top) Stacked bar plots showing proportions of the annotated cell types for WT and *Cdca7*^{G305V} homozygous samples (n=2 biological replicates/genotype). (bottom) Table showing total nuclei numbers retained for analysis for the four samples and nuclei numbers assigned to the different cell types in WT and *Cdca7*$^{G305V/G305V}$ E14.5 cerebrum.

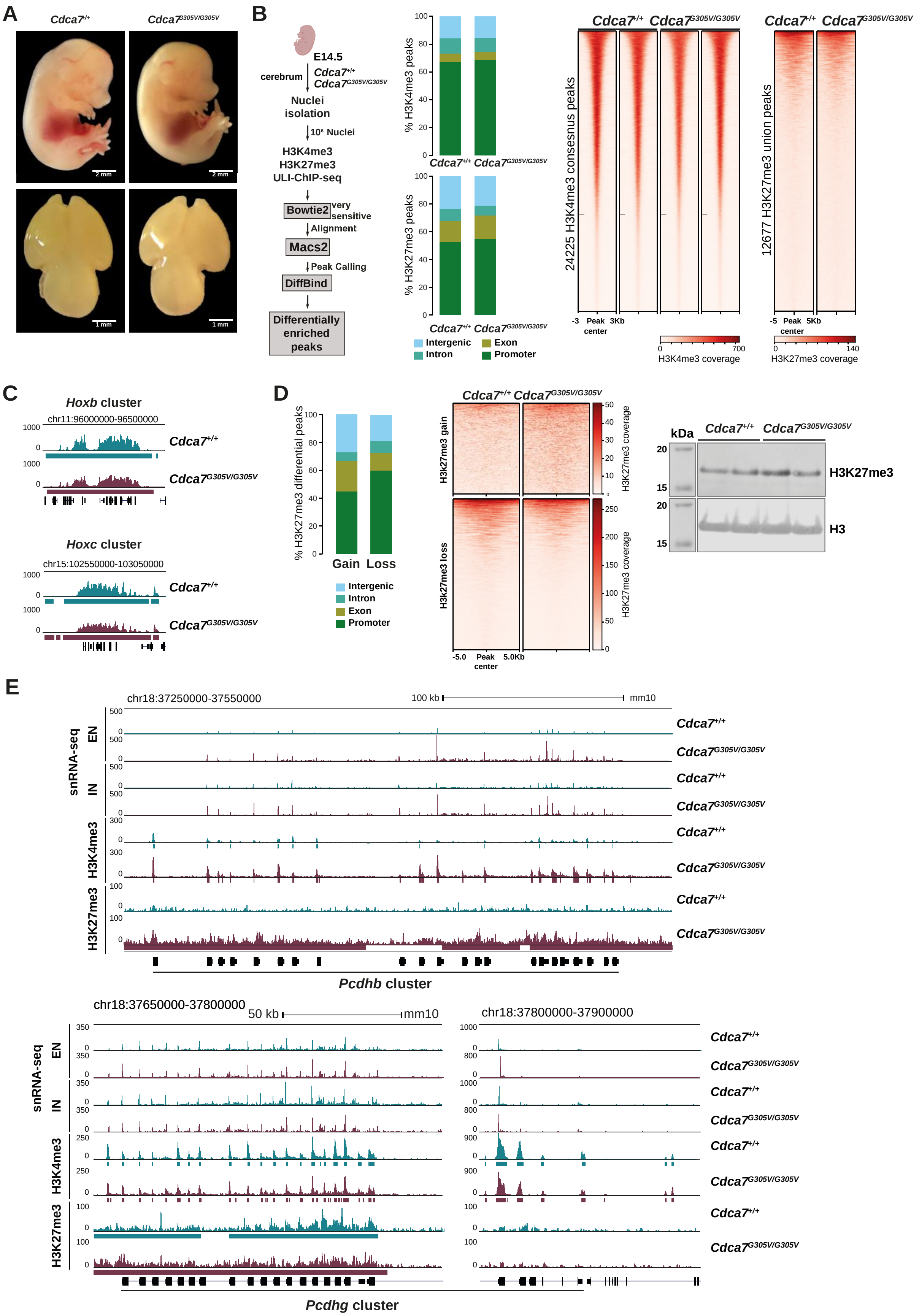
A
Cdca7+/+
Cdca7G305V/G305V
2 mm
1 mm
B
E14.5
cerebrum
Nuclei isolation
10⁶ Nuclei
H3K4me3
H3K27me3
ULI-ChIP-seq
Bowtie2
very sensitive
Alignment
Macs2
Peak Calling
DiffBind
Differentially enriched peaks
% H3K4me3 peaks
% H3K27me3 peaks
Intergenic
Intron
Exon
Promoter
24225 H3K4me3 consesnus peaks
12677 H3K27me3 union peaks
Peak center
H3K4me3 coverage
H3K27me3 coverage
C
Hoxb cluster
chr11:96000000-96500000
Hoxc cluster
chr15:102550000-103050000
D
% H3K27me3 differential peaks
Gain
Loss
H3k27me3 gain
H3K27me3 loss
H3K27me3 coverage
kDa
H3K27me3
H3
E
chr18:37250000-37550000
100 kb
mm10
snRNA-seq
EN
IN
H3K4me3
H3K27me3
Pcdhb cluster
chr18:37650000-37800000
50 kb
chr18:37800000-37900000
Pcdhg cluster

Fig. S12. Dysregulation of clustered *protocadherin* gene expression in *Cdca7*^{G305V} homozygous cerebrum is accompanied by increase of H3K4me3 and H3K27me3.

(A) Representative macroscopic images of E14.5 WT and *Cdca7*^{G305V} homozygous embryos (scale bars, 2 mm) and brains (scale bars, 1 mm).
(B) (left) Schematic representation of ChIP-seq experimental set-up and analysis pipeline (created with BioRender.com). (middle) Stacked bar chart showing proportions of H3K4me3 and H3K27me3 peaks with respect to their genomic annotation. (right) Heatmaps showing H3K4me3 coverage over H3K4me3 peak center (DiffBind consensus peaks) and 3 kb flanking regions. Heatmaps showing H3K27me3 coverage over H3K27me3 peak center (SICER - union peaks) and 5 kb flanking regions.
(C) Genome browser view depicting H3K27me3 profiles over (top) *Hoxb* and (bottom) *Hoxc* clusters. (Rectangles below H3K27me3 tracks – called H3K27me3 peaks; RefSeq annotations are shown below the tracks.)
(D) (left) Stacked bar chart showing proportions of H3K27m3 differential peaks (lost or gained in *Cdca7*^{G305V} homozygotes compared to WT) with respect to their genomic annotation. (middle) Heatmap showing H3K27me3 levels at 6725 differentially enriched peaks (2428 increased and 4297 decreased) in WT and *Cdca7*^{G305V} homozygotes (calculated by SICER, FDR $\leq$ 0.01). (right) Western blot showing H3K27me3 protein levels in E14.5 cerebrum, two biological replicates per genotype. Histone H3 was used as a loading control.
(E) Genome browser screenshots of (top) the 22 clustered *Pcdhb* and (bottom) 21 clustered *Pcdhg* genes. Representative tracks for snRNA-seq (EN - excitatory neurons; IN - inhibitory neurons) and ChIP-seq (H3K4me3 and H3K27me3) from E14.5 cerebrum are shown. (Rectangles below H3K4me3 and H3K27me3 tracks – called peaks; RefSeq annotation is shown below the tracks.)

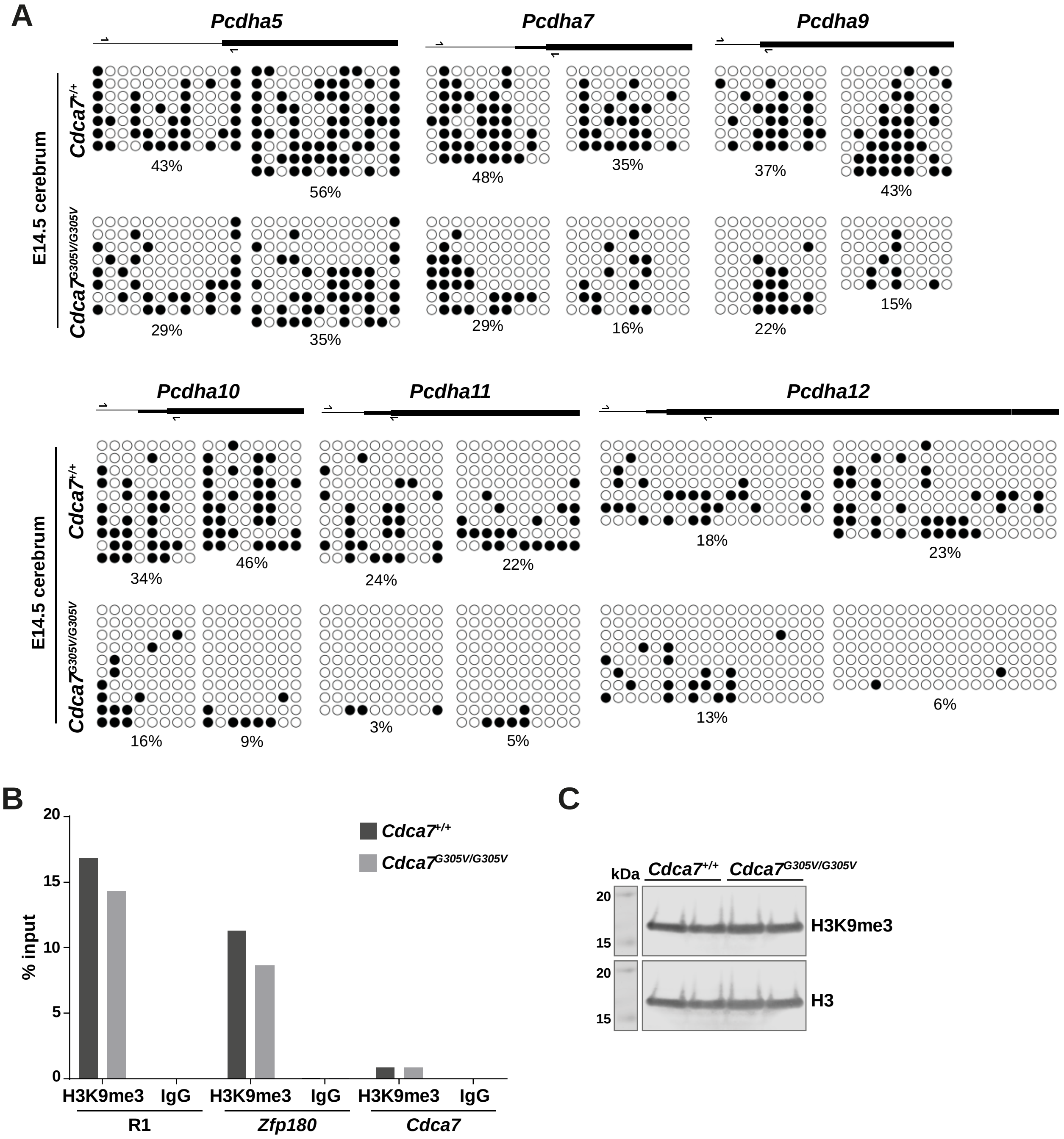

Fig. S13. *Pcdha* locus chromatin marks in WT and *Cdca7*^{G305V} homozygous cerebrum.

(A) DNA methylation levels of *Pcdha2, Pcdha4, Pcdha9, Pcdha10, Pcdha11* and *Pcdha12* promoters were measured by Sanger bisulfite sequencing in WT and *Cdca7*^{G305V} homozygous E14.5 cerebrum (filled circles – methylated cytosines; empty circle – unmethylated cytosines, black box – first exon, black arrows – primers).

(B) ChIP-qPCR showing H3K9me3 enrichment at the R1 site located upstream of the *Pcdha* locus and positive (*Zfp180*) and negative (*Cdca7* promoter) control loci.

(C) Western blot showing H3K9me3 protein levels in E14.5 cerebrum, two biological replicates per genotype. Histone H3 was used as a loading control.

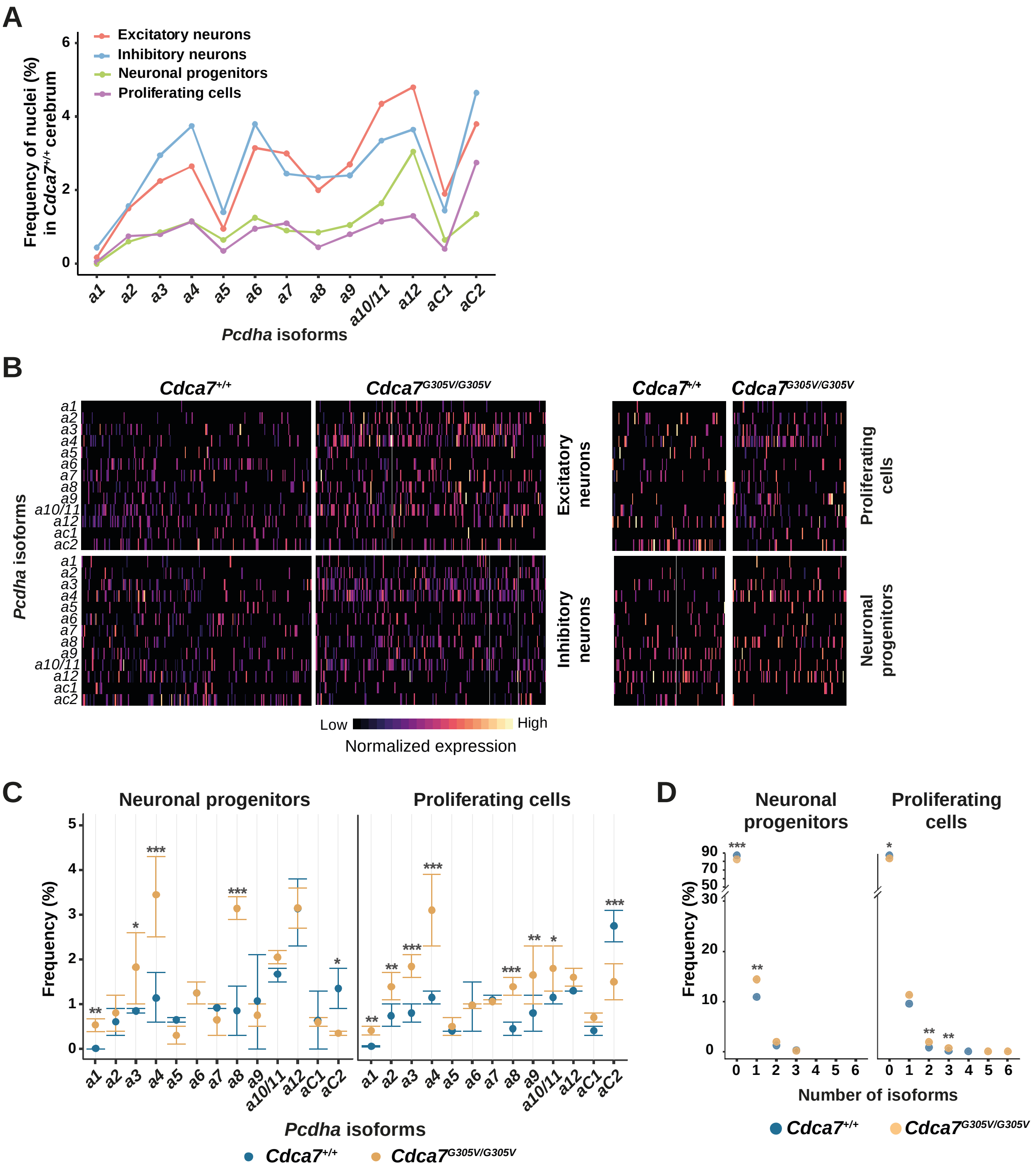

Fig. S14. CDCA7 is a modifier of clustered *protocadherin alpha* stochastic promoter choice. **(A)** Quantification of the frequency of *Pcdha* isoforms expressed in WT single nuclei in the different cell populations. The frequency (in percentage %) shown on the line graph is an average of the two biological replicates; normalized by the total number of nuclei for each cell type (Fig. S11C). Read counts for the *Pcdha10/11* isoforms were combined due to difficulties with Cell Ranger annotation.

(B) Heatmaps showing the normalized expression levels for the 12 variable and 2 constitutive *Pcdha* isoforms in single nuclei. One representative biological replicate per genotype and 200 nuclei for excitatory and inhibitory neurons and 100 nuclei for proliferating cells and neuronal progenitors are shown.
(C) Quantification of *Pcdha* isoform expression frequencies in WT and *Cdca7*^{G305V} homozygous neuronal progenitors and proliferating cells. The frequency (in percentage %) shown on the line graph is the mean +/- standard error of the two biological replicates; normalized by the total number of nuclei for each cell type (Fig. S11C). Read counts for the *Pcdha10/11* isoforms were combined due to difficulties with Cell Ranger annotation. Chi-square test * $p < 0.05$, ** $p < 0.01$, *** $p < 0.001$.
(D) Quantification of the number of *Pcdha* isoforms expressed in WT and *Cdca7*^{G305V} homozygous single nuclei in neuronal progenitors and proliferating cells. The frequency (in percentage %) shown on the dot plot is an average from two biological replicates per genotype; normalized by the total number of nuclei for each cell type (Fig. S11C). Chi-square test * $p < 0.05$, ** $p < 0.01$, *** $p < 0.001$.

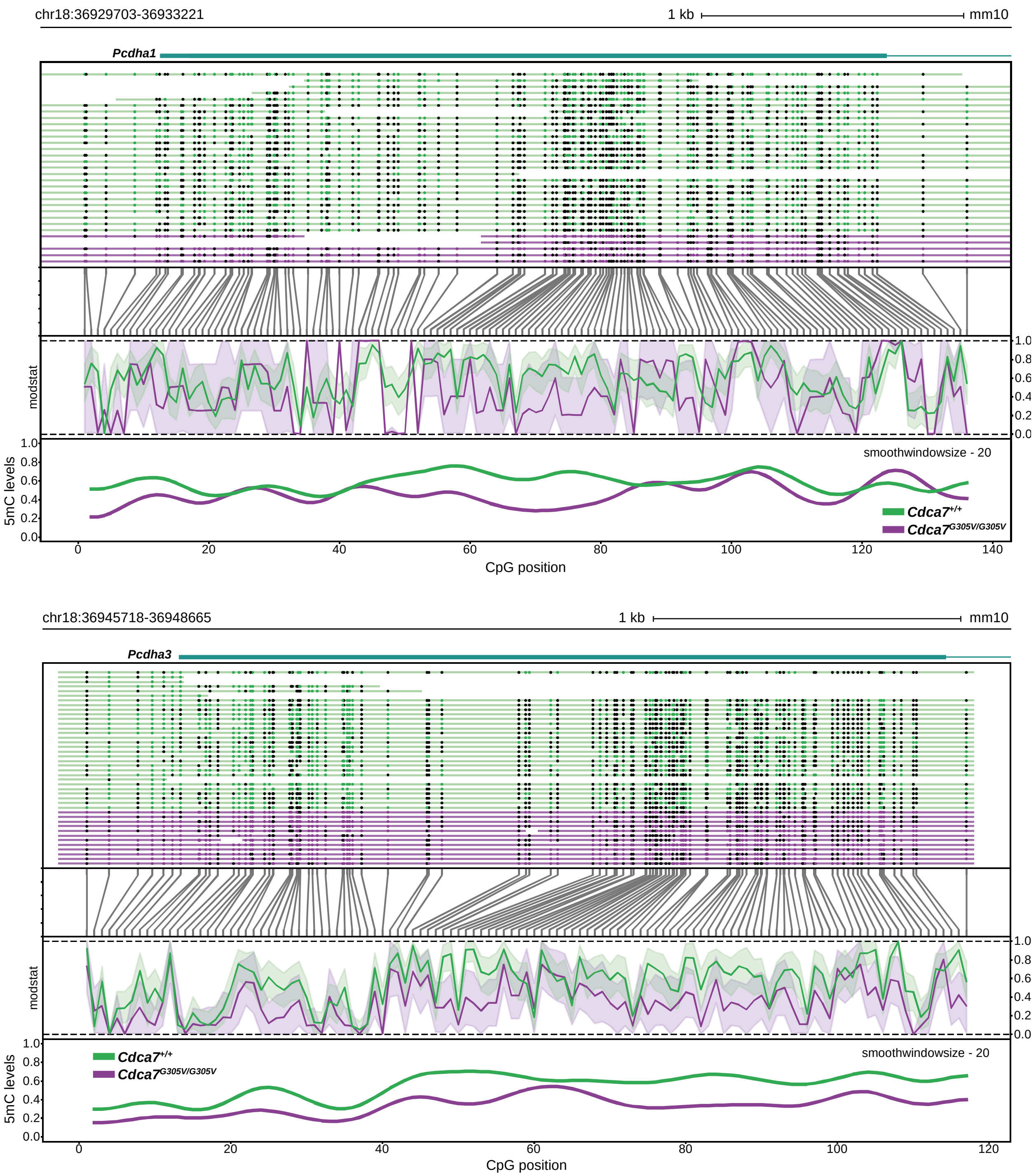

Fig. S15. DNA methylation levels in E8.5 embryos measured by ONT long read sequencing. DNA methylation profile of *Pcdha1* and *Pchda3* promoter regions obtained from ONT long-read sequencing. From top to bottom panels of these figures show i) the genomic position of interest, ii) a diagram showing ONT read alignments, with unmethylated CpGs colored in green (WT) and pink (*Cdca7*^{G305V}), and methylated CpGs colored black, iii) a diagram displaying the correspondence between genome space and CpG space, iv) raw log-likelihood ratios, and v) smoothed methylated fraction plot.

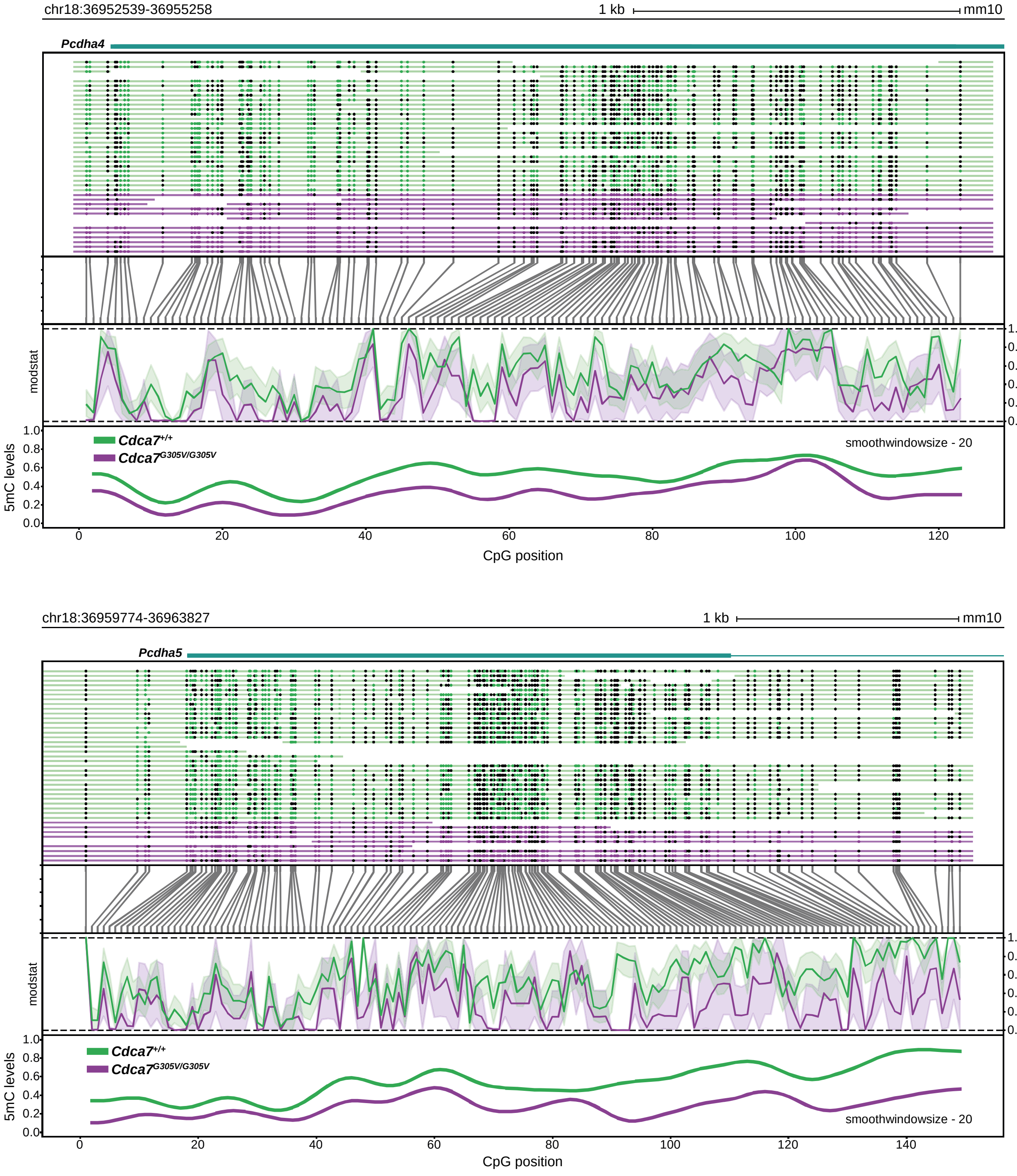

Fig. S16. DNA methylation levels in E8.5 embryos measured by ONT long read sequencing. DNA methylation profile of *Pcdha4* and *Pchda5* promoter regions obtained from ONT long-read sequencing. From top to bottom panels of these figures show i) the genomic position of interest, ii) a diagram showing ONT read alignments, with unmethylated CpGs colored in green (WT) and pink (*Cdca7*^{G305V}), and methylated CpGs colored black, iii) a diagram displaying the correspondence between genome space and CpG space, iv) raw log-likelihood ratios, and v) smoothed methylated fraction plot.

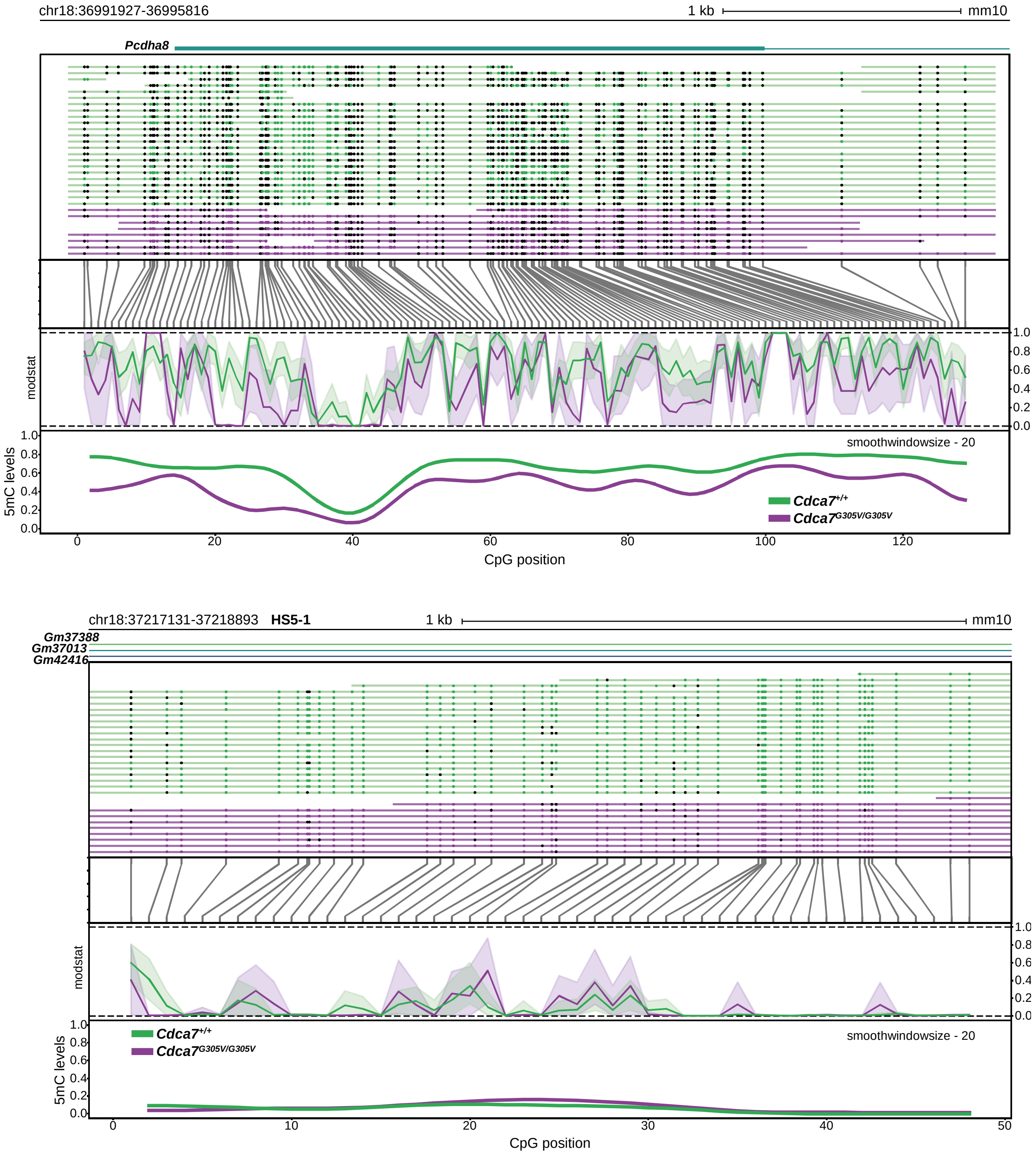

Fig. S17. DNA methylation levels in E8.5 embryos measured by ONT long read sequencing. DNA methylation profile of *Pchda8* promoter region and HS5-1 enhancer obtained from ONT long-read sequencing. From top to bottom panels of these figures show i) the genomic position of interest, ii) a diagram showing ONT read alignments, with unmethylated CpGs colored in green (WT) and pink ($Cdca7^{G305V}$), and methylated CpGs colored black, iii) a diagram displaying the correspondence between genome space and CpG space, iv) raw log-likelihood ratios, and v) smoothed methylated fraction plot.

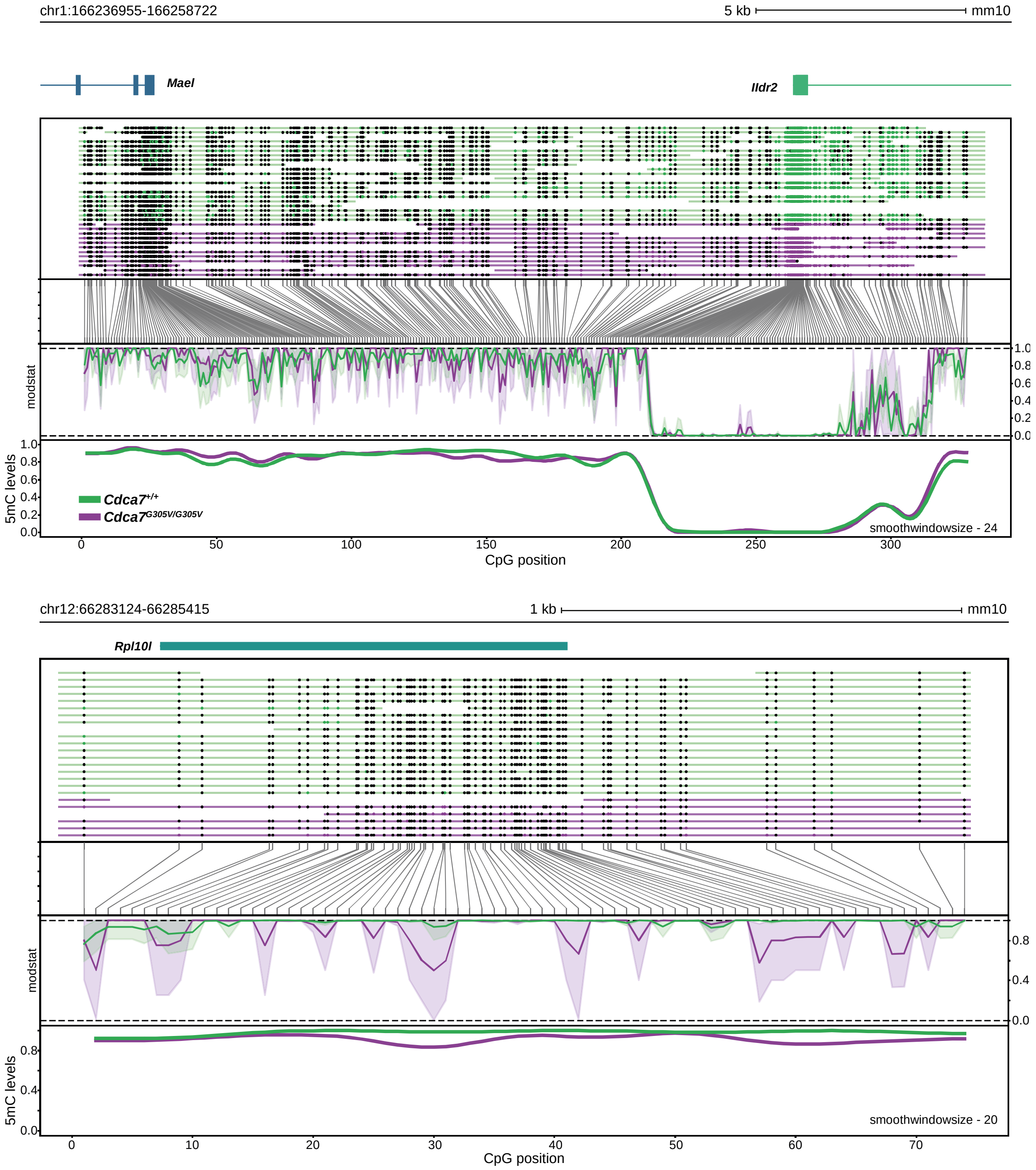

Fig. S18. DNA methylation levels in E8.5 embryos measured by ONT long read sequencing. DNA methylation profile of *Mael* and *Rpl10l* germline genes obtained from ONT long-read sequencing. From top to bottom panels of these figures show i) the genomic position of interest, ii) a diagram showing ONT read alignments, with unmethylated CpGs colored in green (WT) and pink (*Cdca7*^{G305V}), and methylated CpGs colored black, iii) a diagram displaying the correspondence between genome space and CpG space, iv) raw log-likelihood ratios, and v) smoothed methylated fraction plot.

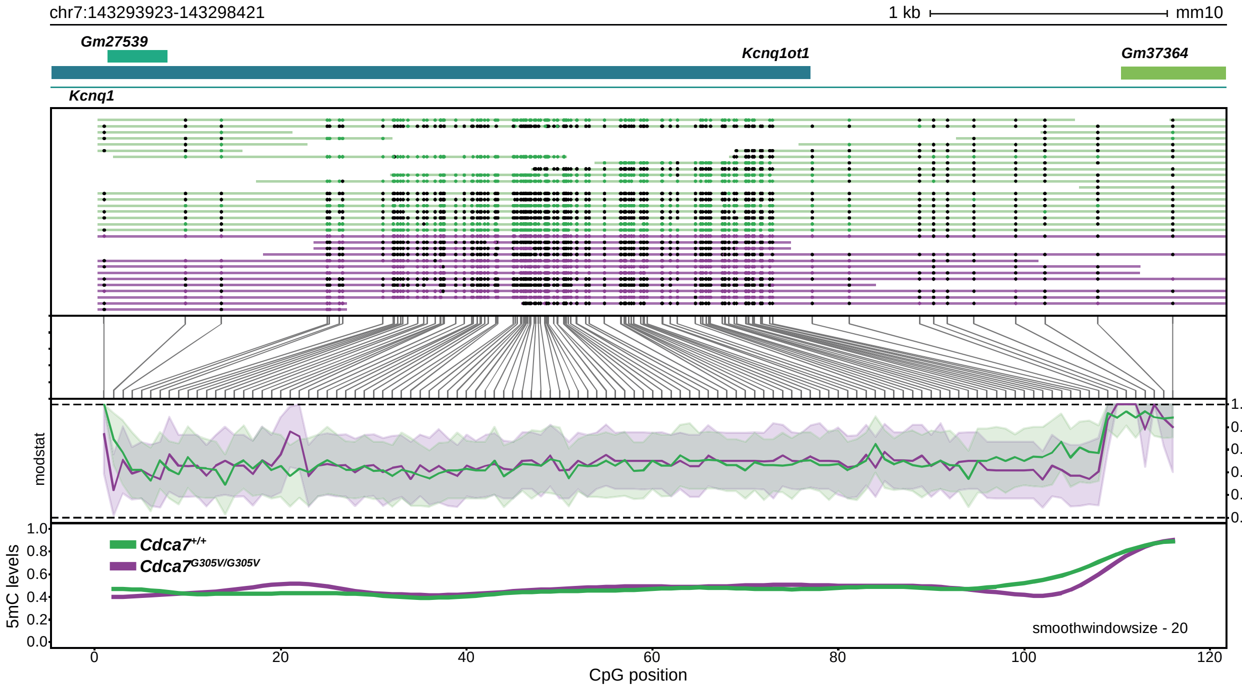

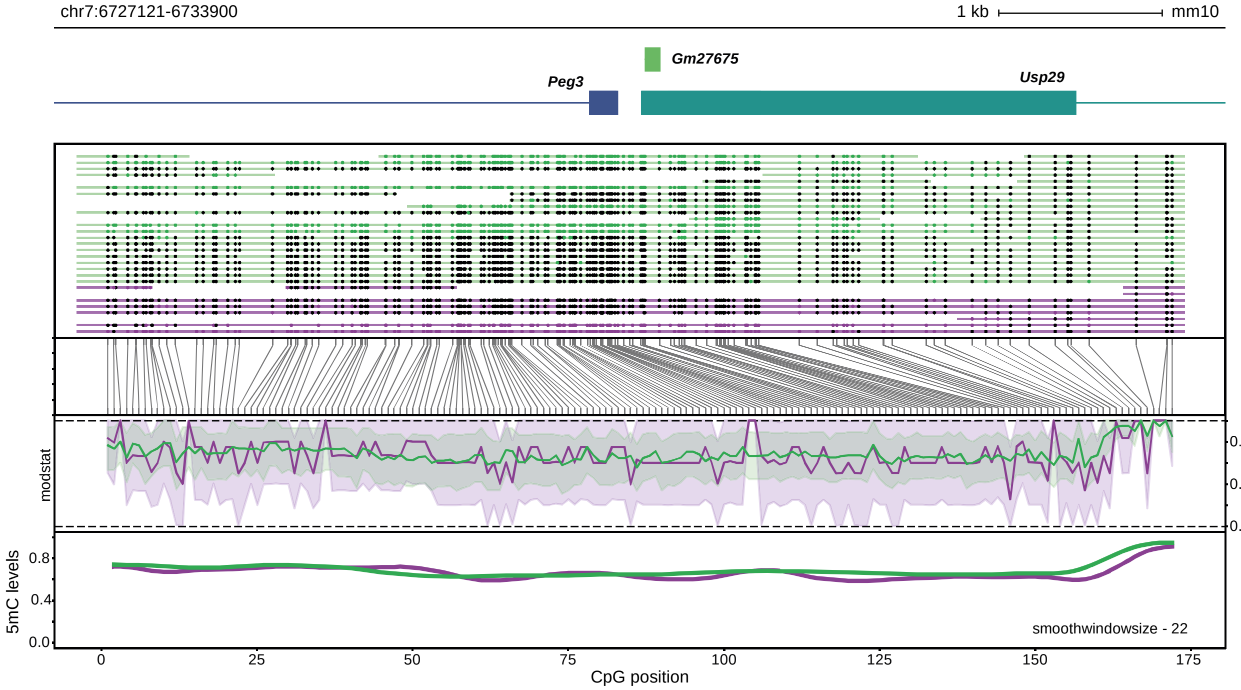

Fig. S19. DNA methylation levels in E8.5 embryos measured by ONT long read sequencing. DNA methylation profile of *Kcnq1* and *Peg3* ICR obtained from ONT long-read sequencing. From top to bottom panels of these figures show i) the genomic position of interest, ii) a diagram showing ONT read alignments, with unmethylated CpGs colored in green (WT) and pink (*Cdca7*^{G305V}), and methylated CpGs colored black, iii) a diagram displaying the correspondence between genome space and CpG space, iv) raw log-likelihood ratios, and v) smoothed methylated fraction plot.

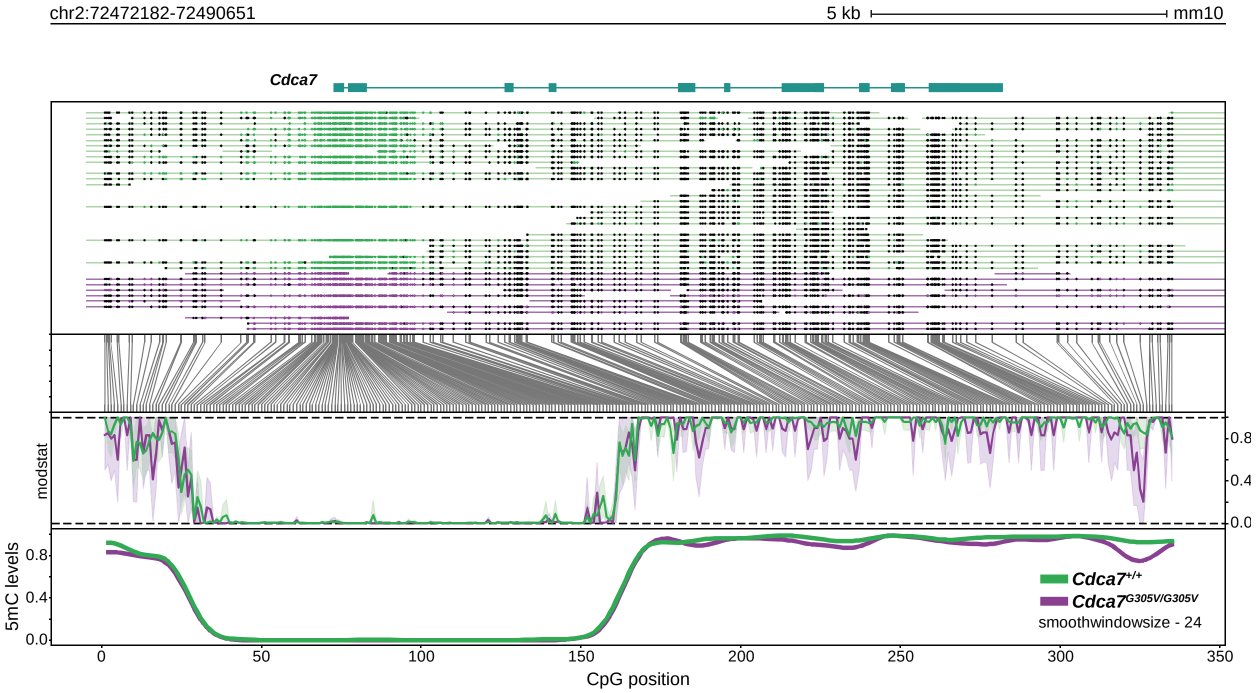

Fig. S20. DNA methylation levels in E8.5 embryos measured by ONT long read sequencing. DNA methylation profile of *Cdca7* gene obtained from ONT long-read sequencing. From top to bottom panels of these figures show i) the genomic position of interest, ii) a diagram showing ONT read alignments, with unmethylated CpGs colored in green (WT) and pink (*Cdca7*^{G305V}), and methylated CpGs colored black, iii) a diagram displaying the correspondence between genome space and CpG space, iv) raw log-likelihood ratios, and v) smoothed methylated fraction plot.

Table S1.

List of primers used in the study.

CRISPR off targets

Off Target Site	Primer sequence (5’ → 3’)		Coordinates (mm10)
OT1 (Retrotransposon, 100% match)	F	CGAAGCCGTGATACTTTGCT	chr16:16015411-16015430
	R	CCGACCCGTAGAAGAAGTCA	
OT2	F	TCTTCTGAGTGTGCCTGTGG	chr4:63295722-63295741
	R	CCAGGCTCTTTCACCAAATC	
OT3	F	TCCGGCTAGGAAGTGAGTTG	chr11:114355052-114355071
	R	ACTTGGCCCACTGAGTCATC	
OT4	F	GGCACTTGGCACAGGTTATC	chr11:101109106-101109125
	R	GCATCTGCTCGTTCCAGAG	

RT-qPCR primers

Primer name	Primer sequence (5’ → 3’)
Cdca7 F	GCAGAACAAAGCAATGCTTG
Cdca7 R	GGGACCTTGACCTGGTAAGA
β-actin F	GGCTGTATTCCCCTCCATCG
β-actin R	CCAGTTGGTAACAATGCCATGT

Southern Blot

Primer name	Primer sequence (5’ → 3’)
Mouse Minor Sat F	CGGCATTGTAGAACAGTGTATATC
Mouse Minor Sat R	CGGTTTCCAACATATGTGTTTTTCAGTG
Mouse Major Sat F	GACCTGTAATATGGCGAGAAAACAG
Mouse Major Sat R	GTCCTTCAGTGTGCATTTCACA
IAP F	TGACATCCTCCTGTGCCATA
IAP R	TTAGCTGGGGATACGTTTGG

Bis-seq primerd

Primer name	Primer sequence (5’→ 3’)	Remarks
Pcdha1 F	GATATATGTATGTAATTGTTAGAGTGGGGTG	(ref 35)
Pcdha1 R1	CAACATTACAAAAATACTACACTTTCC	(ref 35)
Pcdha1 R2	CTATCACACCAAAATCATACTTTACACCTTC	(ref 35)
Pcdha2 F1	AGATTTTGTTGTATTTGAAGAAGTAATA	
Pcdha2 F2	GGTTGAATGGATATATAAAATGTTTTGT	
Pcdha2 R	AAAAACTCTAAATATTTTCTTTCCAAAA	
Pcdha4 F1	ATGTAGTTTTGGTGATTTAAATTTTTAA	
Pcdha4 F2	GGGGAGTTTTTAAATTGTTATAAATTTG	
Pcdha4 R	AAAAATAACTTCTTTTAATCTACCTTCT	
Pcdha5 F	AGTGGTATGTTTATATGTTGTTTTATTT	
Pcdha5 R1	TTCACAAACAAAATACCATTCTACAAAT	

Pcdha5 R2	TAACCTCCTCRAAAATAAAATAATAAAA	
Pcdha7 F1	ATGTAATTATAAAATTAAGGTTTTGGGT	
Pcdha7 F2	GAGATTTTTTTAGGTTTGTTTGTATATT	
Pcdha7 R	ATTATATCCTCTCAAATTTACCATTTCA	
Pcdha8 F1	GGATTAAGTATTTAGGAATGTGGATGATTTTGAG	(ref 35)
Pcdha8 F2	GTTTTTTTAGATATAAGAGAATTGTATTTATGG	(ref 35)
Pcdha8 R	CCTAAACACAACATCTCAAAAACTTAAAATTTCC	(ref 35)
Pcdha9 F1	GGTTTTTATATTTGAAGAAAAAGTTGTA	
Pcdha9 F2	TAAAGATGTGAATTAAAGAAAAAGTGTA	
Pcdha9 R	AAAATACACACAAATTCTAATATCTTCT	
Pcdha10 F1	ATAGTTTGTGTAGTAATATGTTTTTAGATG	
Pcdha10 F2	AGAAGTTTTTAAAGAAAAGTATAAATAA	
Pcdha10 R	CAACAAACACCAAAATTCATAACAATATTTC	
Pcdha11 F1	TAGAATGGTTAATAAATGTGTATTTTGG	
Pcdha11 F2	ATATAAAATGTGAAATTTTTTAGTGGTG	
Pcdha11 R	CCTTCTCTAAAAACTTAACATTACATAT	
Pcdha12 F	TGGAAAATTTAGAAAATGTAAGGGAATAAAGG	(ref 35)
Pcdha12 R1	CATTCTACAAATTTACCTCCAAAAAATCTCC	(ref 35)
Pcdha12 R2	AAACACCAACTCCATCAACTCCAACC	(ref 35)
GFP F1	AAAATAAAATTTTTGGATTGTTATTATTATAA	
GFP F2	ATATTTGTAATTTTAGTATTTTGGGAGGTT	
GFP R	AATCTCTACTCACTACAAACTCCATCTC	

ChIP-qPCR primers

Primer name	Primer sequence (5’→ 3’)
CTCF *Pcdha1* F	ACCCACCAGTTTTCAAAGGC
CTCF *Pcdha1* R	AGTTCGTCTGTTGTCTCTACCT
CTCF *Pcdha2* F	AGGGTGTCGCTGTCTACC
CTCF *Pcdha2* R	AATCCTGCTGCTTCTCCTGT
CTCF *Pcdha3* F	GTTGGAAGGAAATCACAGGTCA
CTCF *Pcdha3* R	ACCGGCTGTTTCTTCTTTGA
CTCF *Pcdha4* F	CCAGGTTCCCAACAACACAA
CTCF *Pcdha4* R	CCAAGACCTTTTACCCGTTCG
CTCF *Pcdha5* F	TCGCTAGACACCACAAGATACA
CTCF *Pcdha5* R	CAGCACCACACAAAGTCCTT
CTCF *Pcdha6* F	AACTTCACTGGGGTTATACGAA
CTCF *Pcdha6* R	GCCATCCTTCAGCAGTCTTG

CTCF *Pcdha7* F	GAGGTGGAGGTGAAGGACAT
CTCF *Pcdha7* R	AGTCAGCAGAGCATTGGAAC
CTCF *Pcdha8* F	GGAGGTAGAGGTGAGGGACA
CTCF *Pcdha8* R	GTCAGCAGAGCATTGGAACC
CTCF *Pcdha9* F	ACATTTAGCCACTGGATGTCG
CTCF *Pcdha9* R	CGAGATCTCCGAACGTAGCT
CTCF HS5-1 F	GCGGCGTTTGGAATCATTTG
CTCF HS5-1 R	TCCAGCACTTTCCTCATCGA
H3K9me3 R1 F	GAAGCGGTGCCACATCTTTT
H3K9me3 R1 R	AGACAAGTGGACACCTCAGG
Zfp180 prom F	AGGGCCAGGATAAATGTAAAACC
Zfp180 prom R	CCTGTGTGGGTTCTTTGGTG
Cdca7 prom F	GTATCCCAGCCTTCCACCTT
Cdca7 prom R	TCTACGCTAAACCCGCAGAT

Data S1. (separate file)

Tables with differentially expressed genes (RNA-seq spleen) and differential peaks (H3K9me3 and H3K27me3 – spleen, H3K4me3 and H3K27me3 - cerebrum) in spleen or cerebrum.

Data S2. (separate file)

Tables in relation to snRNA-seq.
